# Main-Chain Phosphorus-Containing Polymers for Therapeutic Applications

**DOI:** 10.3390/molecules25071716

**Published:** 2020-04-08

**Authors:** Paul Strasser, Ian Teasdale

**Affiliations:** Institute of Polymer Chemistry, Johannes Kepler University Linz (JKU), Altenberger Straße 69, A-4040 Linz, Austria

**Keywords:** polyphosphoester, polyphosphazene, polymer therapeutics, degradable polymers

## Abstract

Polymers in which phosphorus is an integral part of the main chain, including polyphosphazenes and polyphosphoesters, have been widely investigated in recent years for their potential in a number of therapeutic applications. Phosphorus, as the central feature of these polymers, endears the chemical functionalization, and in some cases (bio)degradability, to facilitate their use in such therapeutic formulations. Recent advances in the synthetic polymer chemistry have allowed for controlled synthesis methods in order to prepare the complex macromolecular structures required, alongside the control and reproducibility desired for such medical applications. While the main polymer families described herein, polyphosphazenes and polyphosphoesters and their analogues, as well as phosphorus-based dendrimers, have hitherto predominantly been investigated in isolation from one another, this review aims to highlight and bring together some of this research. In doing so, the focus is placed on the essential, and often mutual, design features and structure–property relationships that allow the preparation of such functional materials. The first part of the review details the relevant features of phosphorus-containing polymers in respect to their use in therapeutic applications, while the second part highlights some recent and innovative applications, offering insights into the most state-of-the-art research on phosphorus-based polymers in a therapeutic context.

## 1. Introduction

Polymers are ubiquitous in medical applications, with polymer-based materials filling a wide range of essential roles, many in everyday use or in clinical translation and with many more in the development pipelines [1]. For example, polymer-based materials can be used in the structural support or replacement of tissues, as ophthalmic devices or dental materials, or in the controlled retention and release of drugs. While historically, off-the shelf polymers have been taken and used as biomaterials (e.g., poly(methyl-methacrylate) (PMMA) for dentistry, polyether urethanes in artificial hearts), this approach also often leads to critical problems in terms of biocompatibility, mechanical properties, and degradation rates; hence, there has been a shift toward specifically designed biomaterials [2]. Synthetic polymers are indispensable in this respect, because their properties can be tuned while offering facile reproducibility and scalability.

With the obvious exception of polydimethylsiloxane (PDMS), all synthetic polymers in widespread clinical use and indeed the vast majority in the research literature utilize organic polymers. However, incorporating main group elements into macromolecules can lead to unique properties that are unachievable with carbon and as such are of increasing interest as carbon-based polymers are pushed to their limits [3]. Phosphorus, being one of the most synthetically versatile and multifaceted elements, comprises a vast number of classes of compounds and is widely applied in fields as far reaching as pharmaceutical to flame retardants and from food additives to pesticides. Nature also chooses phosphorus as a macromolecular building block [4]. Indeed, far from being “the devils element” [5,6], phosphorus is fundamental to life; amongst other functions, adenine triphosphate (ATP) is used to store and transport chemical energy. Furthermore, thanks mainly to its presence in phospholipids, a major component of the cell membrane of all living cells, it is one of the six most abundant elements in living organisms. Of course, DNA, one of the most important biopolymers, is based on a phosphorus main chain, with the multivalent nature of phosphorus as a key feature.

**Figure 1:**
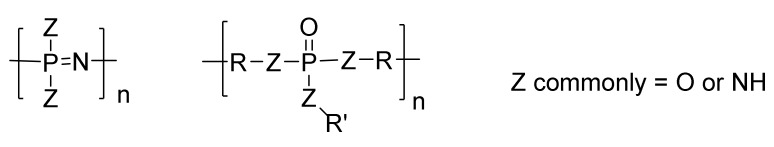
Generic structures for the most common main chain phosphorus-containing polymers polyphosphazenes (PPz) and polyphosphoesters (PPE) and their structural analogues.

In terms of synthetic polymers, there is indeed a substantial research field in which phosphorus exists as a (mostly) pendant group on organic, carbon main-chain, polymers [7]. These polymers are of immense interest for biomedical applications, which is based mostly on the premise of the biomimetic nature of phosphorylcholine-containing (co)polymers [7]. However, these materials are not the focus of this current review, in which we focus on polymers in which phosphorus is an integral part of the macromolecular chains. The most widely investigated polymers with phosphorus in the main chain are the two broad families based loosely around structural analogues of polyphosphazenes (PPz) and polyphosphoesters (PPE), the generic structures for which are shown in Figure 1. In recent decades, a better fundamental understanding of the structure–property relationships for these polymer families has been combined with vast improvements in controlled polymerization methods. This has allowed for the preparation of materials with evermore finely tuned macromolecular structures, and with it, more advanced and precise tailor-made properties. The chemical versatility leads to a wide variety of structures and properties and hence the fields of application are similarly wide, ranging from high-performance elastomers to flame retardants [8]. Herein, we place our attention on recent developments in phosphorus-based polymers for therapeutic applications and detail the relevant structural and chemical features.

## 2. Macromolecular Structural Features of Main-Chain Phosphorus-Containing Polymers

### 2.1. Synthesis

#### 2.1.1. Traditional Synthesis Routes

The traditional and still most prevalent route to prepare high molecular weight PPz is via the ring-opening polymerization of the cyclic trimer [NPCl_2_]_3_ to give [NPCl_2_]*_n_*. This reaction is usually carried out under vacuum at high temperatures: typically 250 °C for several hours. The polymerization can be catalyzed with Lewis acids (e.g., AlCl_3_) to lower the initiation temperature to around 200 °C or HSO_3_(NH_2_) conducted in refluxing trichlorobenzene [9], or alternatively prepared directly from phosphorus pentachloride (PCl_5_) and ammonium chloride via an in situ formation of the cyclic trimer [9]. While the [NPCl_2_]*_n_* precursor is highly sensitive to hydrolysis, it can be stabilized, characterized, and stored in diglyme [10]. The highly labile nature of the P-Cl bonds along the main chain of [NPCl_2_]*_n_* allows for their facile functionalization, and large libraries of poly(organo)phosphazenes have been prepared [11]. Meanwhile, the traditional route to polyphosphoesters is the step-growth polymerization of a dichlorophosphate with diols. Copolymerization with a wide range of aromatic or aliphatic diols is straightforward, although sometimes harsh reaction conditions (temperatures up to 300 °C, vacuum) are required, and acidic condensation products must be removed [12]. Due primarily to the availability of the monomers, these traditional routes to both polyphosphoesters and polyphosphazenes remain the commercially most viable. However, for both mechanisms, it is inherently difficult to control the molecular weight distributions, and while polydisperse materials can be tolerated for some, but not all applications, polymer chemists have sought to develop more controlled routes [13].

#### 2.1.2. Controlled Polymerization Routes

**Figure 2:**
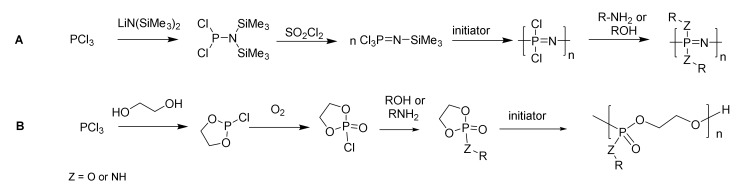
Examples of controlled polymerization routes to polyphosphazenes (**A**) and polyphosphoesters (**B**).

Many modern applications, especially in highly regulated pharmaceutical fields, demand control and reproducibility over molecular weight (MW) and dispersity (Ð). This is especially important for applications in solution, such as for example polymer therapeutics, where the size of the polymers plays a crucial role in their biodistribution. Well-controlled methods, starting from PCl_3_, have been developed for both polyphosphazenes and polyphosphoesters, as shown in Figure 2.

**Figure 3:**
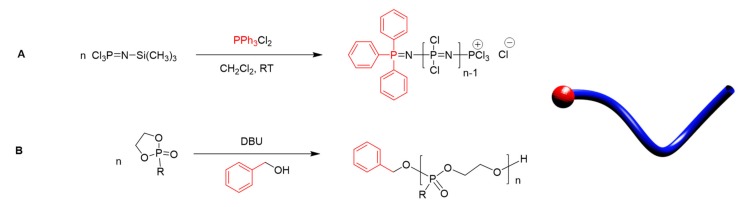
Monodirectional growth for chain-end functionalized polyphosphazenes (**A**) and polyphosphoesters (**B**).

The most used controlled synthesis for [NPCl_2_]*_n_* involves the living cationic polymerization of trichlorophosphoranimine (Cl_3_PNSiMe_3_), which is initiated with PCl_5_ [14,15]. The reaction proceeds readily at ambient temperatures, and the M_n_ can be controlled by the ratio of PCl_5_ to Cl_3_PNSiMe_3_, with control maintained up to chain lengths in the region of n≈50–100. This can be carried out in one pot directly from PCl_3_ (Figure 3A), hence without tedious purification of the intermediates [16], which is suitable for upscaling, albeit with some loss of control. More recently, it has been shown that starting from Ph_3_PCl_2_, monodirectional growth can be ensured [17], as shown in Figure 3B, which is a feature that opens the door to the development of chain-end functionalized polyphosphazenes [18], as well as higher architectures such as polyphosphazene block copolymers [19], block copolymers with organic macroinitiators [20], and star-branched and bottle-brush polyphosphazenes [21].

Similarly, the development of controlled routes to polyphosphoesters has led to a recent surge in their development [22]. The controlled synthesis of polyphosphoesters can be achieved by the ring-opening polymerization (ROP) of cyclic phospholane monomers in a reaction analogous to the ROP of polylactides [22]. This reaction can be initiated either with metallic catalysts, such as stannous octoate (Sn(Oct)_2_) or strong organic bases, most commonly DBU (1,5-diazabicyclo[5.4.0]undec-5-ene) [23]. The latter route is of importance for biomedical applications due to the avoidance of toxic metal catalysts. The use of a monofunctional alcohol to cap the chain end can be used to achieve control of the end groups [23] (Figure 3B), increasing the control of the synthesis and allowing the preparation of block copolymers via organic macroinitiators. The inherent necessity for ring strain limits the ROP to cyclic phospholane monomers of certain ring sizes, with the five-membered ring being the most commonly applied monomer. Generally, a high MW and low Ð (up to 100,000 g mol^−1^, Ð < 1.10) can be achieved using this method, thus circumventing a number of problems commonly associated with organic polyesters, such as transesterification, which can lead to broader Ð values [22].

#### 2.1.3. Alternative Controlled Polymerization Routes

**Figure 4:**
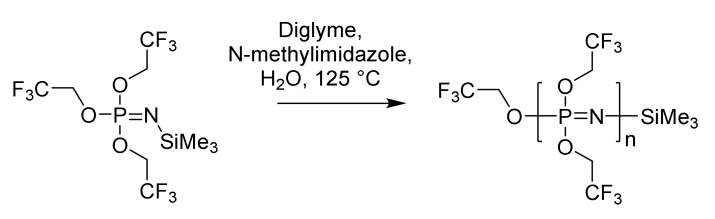
Controlled synthesis of poly(bis(2,2,2-trifluoro-ethoxy)phosphazene) from the respective phosphoranimine.

Controlled polymerization of the relatively accessible monomer trifluoroethoxyphosphoranimine is also possible, resulting in polymers with narrow Ð values of approximately 1.15 for an M_n_ of 25 KDa (Figure 4) [24]. It is thought that rapid initiation by traces of H_2_O produces a phosphazene anion that propagates monodirectional growth at the TMS (trimethylslilyl) end of the polymer. This method, while thus far limited to fluoroethoxy-substituted polyphosphazenes and relatively low MW, is important as it allows controlled, multigram-scale synthesis of this clinically important polymer (see Section 4.1).

**Figure 5:**
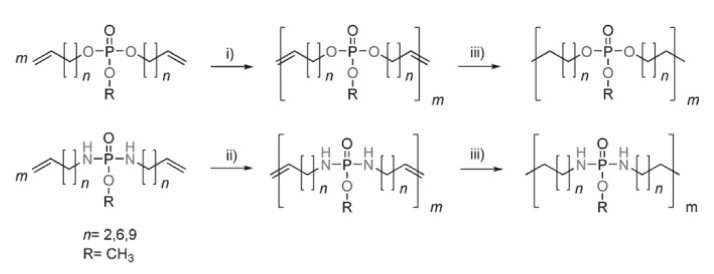
Acyclic diene metathesis (ADMET) polymerization of unsaturated phosphates and phosphorodiamidates: (**i**) Grubbs first generation catalyst, 50 °C, bulk; (**ii**) Grubbs–Hoveyda second-generation catalyst, RT, 1-chloronaphthalin; (**iii**) Pd/C, RT, CH_2_Cl_2_. Reproduced from [27], with permission from John Wiley and Sons.

Olefin metathesis polymerization offers an alternative and promising route to polymers with phosphorus in the main chain, making use of the high functional group tolerance of modern metathesis catalysts. Efficient methods utilizing readily available and easy to handle monomers have been developed for the ring-opening metathesis (ROMP) [25] and acyclic diene metathesis (ADMET) polymerization [26] for the preparation of both phosphoesters and phosphoamidates (Figure 5) [27].

#### 2.1.4. Polymers with Phosphorus–Carbon Bonds

**Figure 6:**
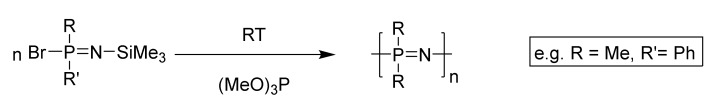
Synthesis of poly(alkyl/aryl)phosphazenes in a controlled manner from bromophosphoranimines.

Polymers with substituents attached via P-C bonds, rather than the P-N or P-O discussed above, tend to be more resilient to chemical hydrolysis. This applies to poly(alkyl/aryl)phosphazenes [28], which can be prepared in a controlled manner from bromophosphoranimines (Figure 6) with over 40 variants reported with different combinations of a variation of aliphatic and aromatic substituents. While chemical stability is an advantage in terms of synthesis, these polymers tend to be hydrolytically inert and hence suited to such applications where biostability is required, rather than biodegradability [28].

**Figure 7:**
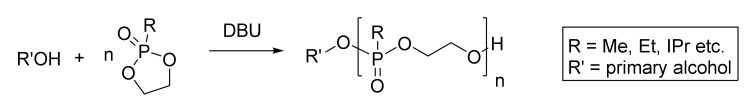
Poly(alkyl ethylene phosphonate)s prepared via DBU (1,5-diazabicyclo[5.4.0]undec-5-ene) -catalyzed ring-opening polymerization (ROP).

Similarly, poly(alkyl ethylene phosphonate)s (Figure 7) are more stable toward hydrolysis than the corresponding polyphosphates. However, they can still undergo hydrolysis via the P-O bonds in the backbone. The stability of the P-C bond reduces the tendency to transesterification, which can be problematic during the synthesis of polyphosphoesters (see Section 2.1.2). Poly(alkyl ethylene phosphonate)s can be prepared by the DBU-catalyzed ROP with Ð values of approximately 1.1 up to an M_n_ of 20 KDa [29]. In contrast to polyalkylphosphazenes, these polyalkylphosphonates can also be water soluble and hydrolytically degradable [30].

#### 2.1.5. Post-Polymerization Functionalization

The post-polymerization functionalization of macromolecules is an attractive feature as it can be used to further enhance functionality, without preparing a new monomer, foregoing the extensive process optimization that this generally entails. Post-polymerization functionalization is particularly attractive in designing polymers for therapeutic applications, since it allows facile loading and labeling, as well as tuning properties such as solubility and sol–gel transition (see Section 2.4.3). The ease of substitution of labile P-Cl moieties in macromolecular precursors facilitates this. For example, for PPz, the highly reactive [NPCl_2_]*_n_* precursor can be replaced with a host of nucleophiles in a process often referred to as macromolecular substitution. P-H or P-Cl moieties in polyphosphoesters can similarly be used to undertake post-polymerization modification.

**Figure 8:**
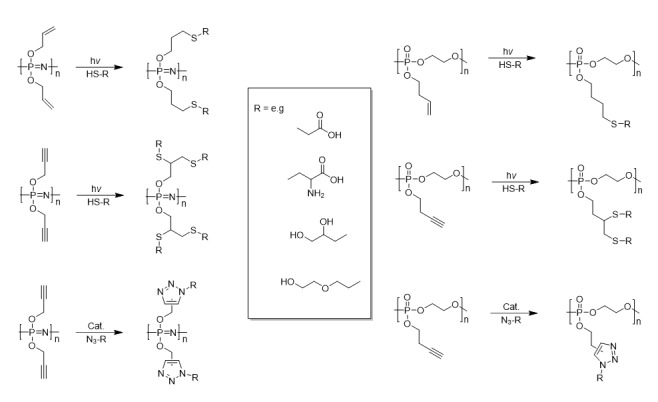
Exemplary structures for the post-polymerization functionalization via thiol-ene addition and Huisgen cycloaddition of azides onto phosphorus-containing polymers with unsaturated substituents.

For efficient, clean post-polymerization functionalization, “clickable” strategies have been developed via phosphorus atoms bearing pendant unsaturated carbon moieties. Such strategies avoid the use of protecting groups and thus further expand the functional groups available to allow for those not compatible with halogen replacement (such as strong nucleophiles and acids) for PPz and the ring-opening polymerization in the case of PPE [31], which is similarly intolerant to nucleophiles and acids. Figure 8 shows some exemplary strategies for the alkoxy-derived poly(organo)phosphazenes [32] and polyphosphoesters [33], while analogous systems with amine derived substituents can also be prepared [34,35]. Furthermore, for some of the polymers, for example those derived from cyclic phospholanes [33] as well as poly[bis(allylamino)phosphazene] based systems [36], the reactions can be carried out in aqueous solutions. The alkyne-functionalized polymers can similarly be used for the Huisgen cycloaddition of azides for both polyphosphoesters [33] as well as polyphosphazenes [37].

### 2.2. Macromolecular Architecture

A variety of highly branched polymers, for example hyperbranched, dendritic, star, and bottle-brush polymers, are available for phosphorus-containing polymers, due in part to the controlled polymerization methods described in Section 2.1.2 [21]. This development is driven partly due to the high functionality and large number of end groups for branched polymers and the better solubility and lower viscosity compared to their linear counterparts. Such properties are particularly relevant for solution-based applications such as drug delivery [38], and indeed macromolecular architecture is also understood to have an essential role in biodistribution [39].

**Figure 9:**
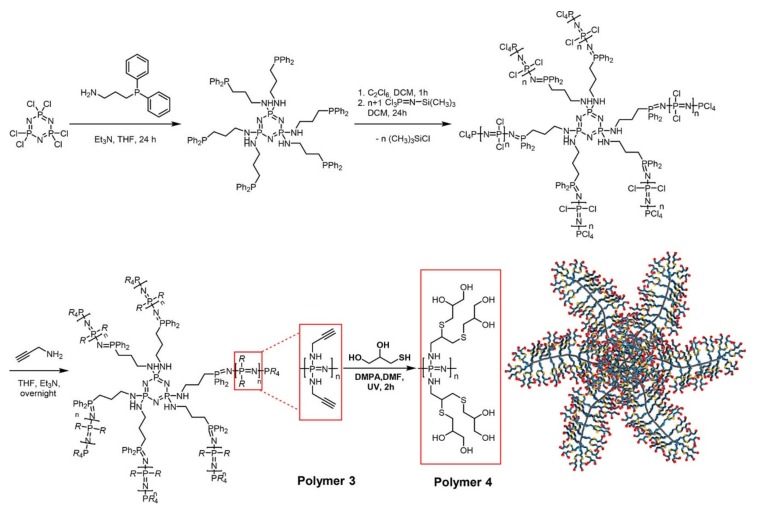
Synthetic route to star-branched PPz via a combination of phosphine-mediated polymerization from a hexafunctional core with thiol–ene post-polymerization functionalization to produce highly branched polyols. Reproduced from [42] (https://pubs.acs.org/doi/abs/10.1021/acs.iecr.7b05301), with permission from the American Chemical Society.

Water-soluble bottle-brush-type architectures are readily available for polyphosphazenes, for example by grafting organic polymers either to [32] or from [40] the two available P-Cl moieties per repeat unit. Furthermore, polyphosphazenes can also be grafted from polymers bearing arylphoshines as initiating points to generate star-branched and brush architectures [41]. For example, a combination of this with thiol-yne post-polymerization functionalization has been used to prepare highly branched polyols (Figure 9) [42].

**Figure 10:**
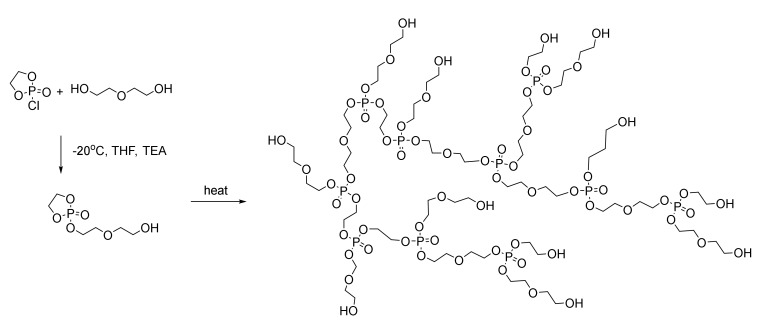
AB*-type inimer approach to the preparation of hyperbranched polyphosphoesters.

A highly useful, simple one-pot AB*-type inimer approach to prepare hyperbranched polyphosphoesters has been developed (Figure 10) that combines the ROP route to PPE by preparing monomers from 2-chloro-2-oxo-1,3,2-dioxaphospholane (COP) with a free hydroxyl group from diethylene glycol to give an AB*-type inimer [43]. Upon mild heating and in the absence of catalysts, the primary hydroxyl in one AB*-type inimer molecule triggers the ring-opening reaction of the five-membered phosphate ring in another AB* molecule and leads to the formation of two primary hydroxyls. Further repetitions lead to high molecular weight, water-soluble hyperbranched polymers. Furthermore, the hydroxyl end groups can be utilized as macroinitiators, for example, for the growth of polyphosphate arms or for polyethyleneglycol (PEG)ylation [44].

**Figure 11:**
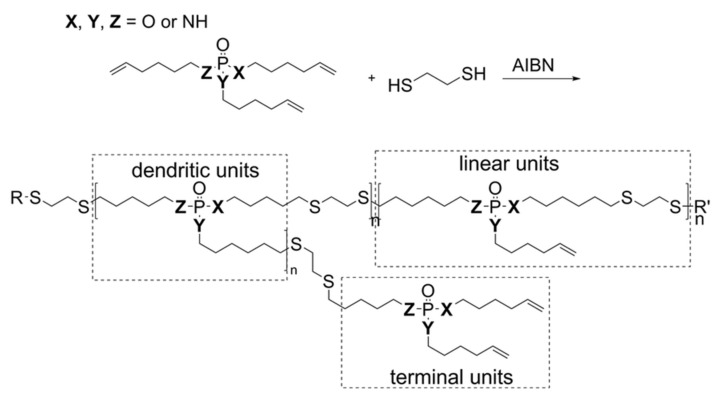
Unsaturated organophosphates and their polymerization via thiol–ene addition chemistry to hyperbranched polymers. Adapted from [45]; published by The Royal Society of Chemistry.

Wurm and coworkers have also successfully developed simple one-pot polymerization methods to prepare hyperbranched polyphosphoesters from phosphate-based trienes, which themselves are readily accessible from POCl_3_ (Figure 11). Such alkene-bearing phosphates can be polymerized either via thiol–ene addition chemistry [45] or by acyclic triene metathesis (ATMET) using established Grubbs catalysts [46].

### 2.3. Dendrimers

**Figure 12:**
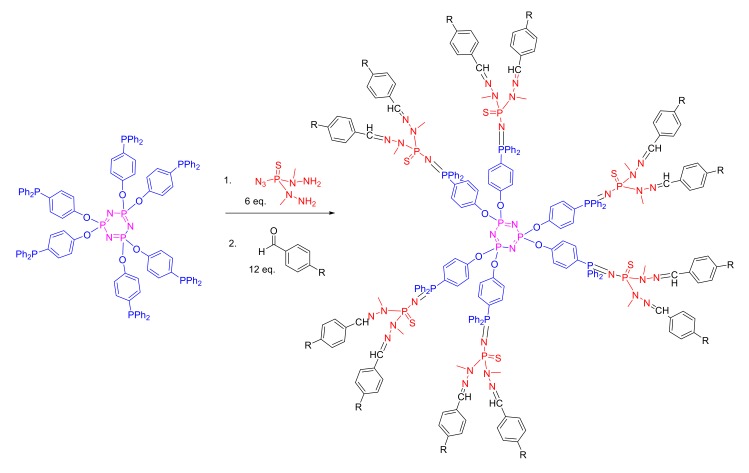
The first generation of an “onion peel” phosphorus-based dendrimer [50].

A broad range of well-defined, monodisperse dendrimers based on phosphazene chemistry can be prepared utilizing the high functionality efficient chemistry at the phosphorus center [47,48,49]. Figure 12 shows an elegant example of polymers that represent the pinnacle in terms of size-controlled syntheses [50]. With an arylphosphine-functionalized phosphazene trimer forming the hexafunctional core, azides can be added via an atom-efficient Staudinger reaction with N_2_ gas as the only side product. In the example shown, azidothiobishydrazinophosphine is added, bearing two methylhydrazino groups. The resulting P=N-P=S is more stable than the more obvious phosphine oxide. Then, orthogonal Schiff-base chemistry adding aldehydes to the hydrazide moieties is used to add further generations or functional groups. Upon the functionalization of organophosphates, these dendrimers contain no less than seven types of phosphorus, demonstrating the versatility and diversity of incorporating phosphorus into macromolecular structures.

### 2.4. Amphiphilic Polymers

#### 2.4.1. Micelle Formation

For nanomedicine, the self-assembly of nanoformulations and nanoaggregates, such as micelles or polymersomes, is important as this can increase the size to the 50–100 nm range and facilitate controlled release pathways. The controlled synthesis of the macromolecular building block facilitates these processes, allowing the preparation of block copolymers with defined block sizes. Furthermore, post-polymerization functionalization can be used to adjust the lipophilicity and or charge of the chains, thus manipulating the aggregation process into nanoaggregates.

**Figure 13:**
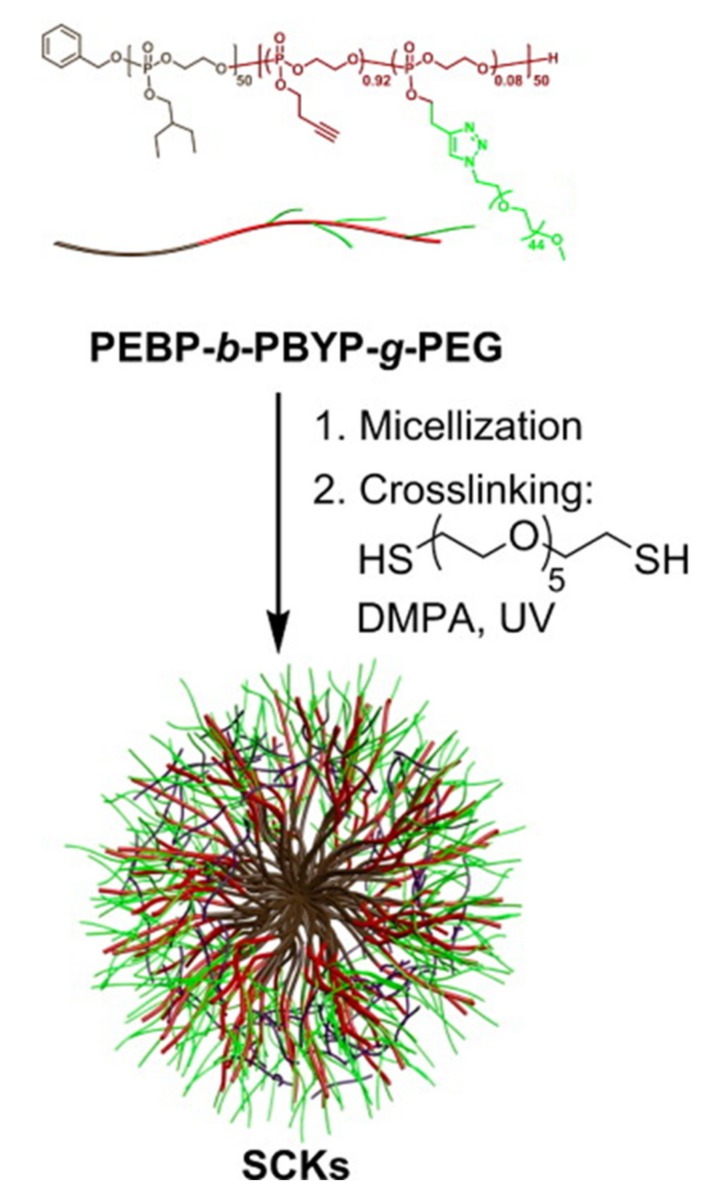
Shell cross-linked knedel-like nanoparticles (SCKs) prepared via an amphiphilic-functional AB diblock polyphosphoesters. Alkyne moieties could not only be functionalized with polyethyleneglycol (PEG) oligomers to form the hydrophilic exterior, but they could also be used for shell cross-linking with dithiols to give stable nanoparticles. Adapted with permission from [53]. Copyright (2015) American Chemical Society.

The monodirectional ROP of cyclic phospholanes enables the facile synthesis of block copolymers of polyphosphoesters. Should these two blocks consist of hydrophobic and hydrophilic sections, then they will tend to self-assemble into micelles in aqueous environments. For example, simple amphiphilic block copolyphosphates from poly(2-ethoxy-2-oxo-1,3,2-dioxaphospholane)-*block*-poly(2-isopropoxy-2-oxo-1,3,2-dioxapholane) (PEP-*b*-PIPP) have been developed [51]. Wooley et al. have used these to prepare sophisticated stable shell cross-linked knedel-like nanoparticles (SCKs) (Figure 13) [52]. An amphiphilic-functional AB diblock polyphosphoester was synthesized with hydrophobic 2-ethylbutoxy phospholane as a hydrophobic section, plus 2-butynyl phospholane (BYP) [53]. The alkyne groups could not only be functionalized with PEG oligomers but also used for shell cross-linking with dithiols to give stable SCKs, which are being investigated as drug carriers (see Section 3.1.2).

**Figure 14:**
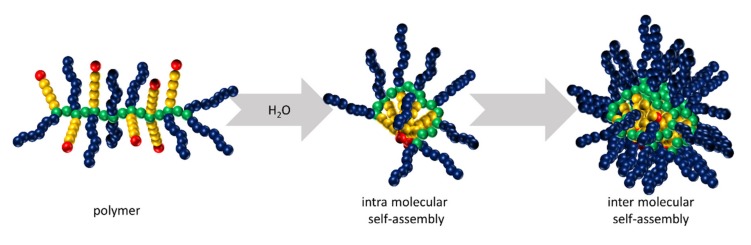
Amphiphilic polyphosphazene with a statistically distributed array of hydrophobic and hydrophilic substituents undergoes self-assembly in aqueous solutions. Reproduced with permission from [55].

A simple post-polymerization functionalization of PPzs with different ratios of a hydrophobic and hydrophilic substituents is also widely used to form nanoaggregates [54]. The unique conformational flexibility of the polyphosphazene main chain allows aggregation of the hydrophobic moieties and thus the formation of micelle-like structures in the nanosize region (Figure 14) [54].

#### 2.4.2. Polymersomes

**Figure 15:**
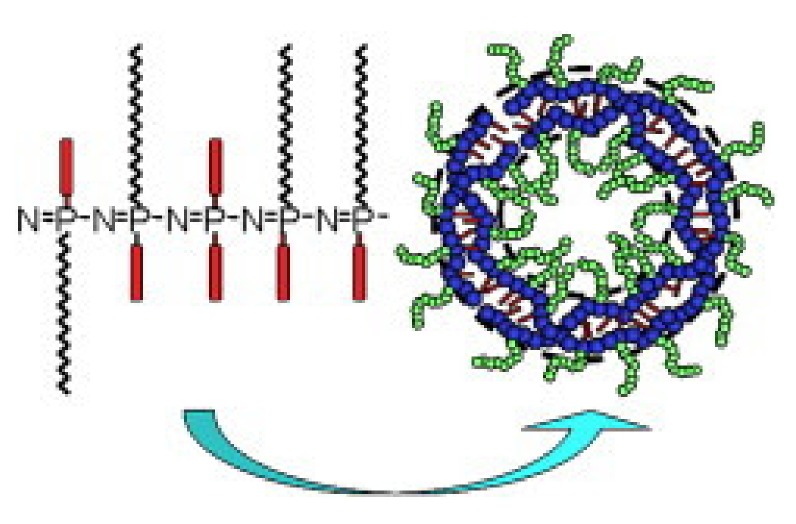
Stable polymersomes formed from amphiphilic, randomly substituted PPz. Reproduced from [56], with permission from Elsevier.

As with organic macromolecules, the ratio of hydrophobic to hydrophilic segments determines to a large extent the structure of self-assembled systems. It has been widely demonstrated that grafted polyphosphazenes can also form stable polymersomes (Figure 15) [56]. Qiu and coworkers established self-assembly rules for grafted polyphosphazenes and found that they possess a regularity of structure transitions allowing the design of polyphosphazene vesicles. The higher stability compared to liposomes renders them ideal drug carriers [54].

**Figure 16:**
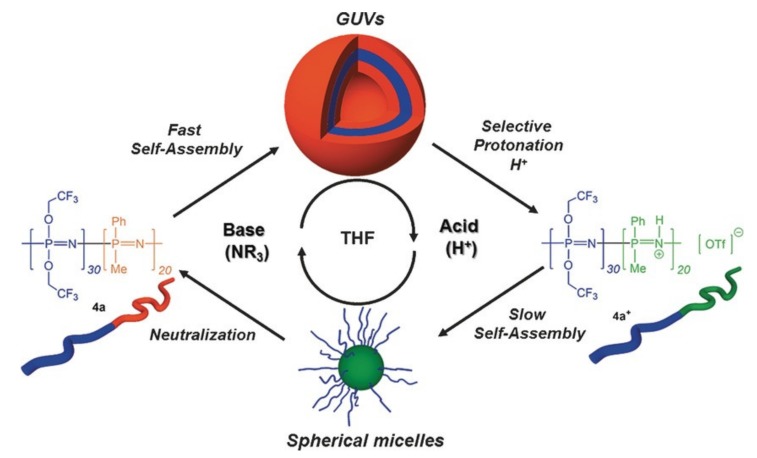
Crystalline-*b*-coil block copolyphosphazenes undergo self-assembly to giant unilamellar vesicles (GUVs) in THF. Upon protonation/deprotonation, a reversible switching of the morphology to small spherical micelles is observed. Reproduced from [57], with permission from John Wiley and Sons.

Furthermore, Soto and coworkers have developed, amongst other nanomorphologies [19], giant polymersomes, so-called giant unilamellar vesicles (GUVs) with good stability over a long time range [57]. Giant polymersomes (approximately 10–100 μm) and GUVs (>1 μm) as opposed to standard polymersome sizes in the region of 100 nm diameters are of special interest due to their similar size to cells making them better suited to the biomimicry of cellular processes. The self-assembly of crystalline-*b*-coil block copolyphosphazenes is fast and additive-free, albeit in nonaqueous solvents (THF). Furthermore, the GUVs protonation/deprotonation leads to a reversible switching of the morphology to small spherical micelles in which the core/corona roles are inverted (Figure 16). Copolymerization with non-polyphosphazene blocks also leads to other self-assembled nanomorphologies, including that of bicontinuous nanospheres or toroidal micelles [19].

Although the preparation of polymersomes has been relatively less frequently described for polyphosphoesters, they have also been reported, such as for example poly(ɛ-caprolactone)-block-poly(ethyl ethylene phosphate), in which the polyphosphate forms the hydrophilic part [58]. However, of particular interest is the recently reported amphiphilic block copolymers polybutadiene-*block*-poly(ethyl ethylene phosphate) (PB-*b*-PEEP), with a polyphosphoester as the hydrophilic segment. These were superior at forming GUVs in aqueous dispersion when compared to organic polymers commonly used for this purpose and could be formed by a simple film hydration process [59].

#### 2.4.3. Thermosensitive Polymers

**Figure 17:**
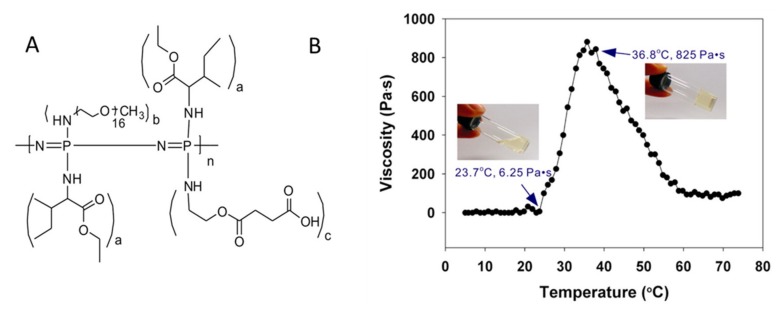
(**A**) Common structure for a thermosensitive polyphosphazene. Ratio of a to b groups can be used to fine-tune the lower critical solution temperature (LCST), whilst c groups are used for adding functionality, for example drug conjugation. (**B**) Exemplary temperature-dependent sol–gel transition and viscosity change of 13 wt % aqueous solution of a typical isoleucine–PEG polyphosphazene. Adapted with permission from [72]. Copyright (2012) American Chemical Society.

Amphiphilic polymers often undergo lower critical solution temperature (LCST), or the opposite upper critical phase separation (UCST) transitions. This is no different for polymers with phosphorus in the main chain. When the transitions are in a biologically relevant temperature range, they can be used for responsive biomedical materials, such as drug release or injectable hydrogels. Indeed, a wide range of such polyphosphazenes and polyphosphoesters has been reported. The dual functionality and post-polymerization functionalization of polyphosphazenes facilitates facile tuning of the LCST temperature [60] in order to achieve biologically relevant temperatures. While other applications have also been investigated, e.g., nanomotors [61], the most widely studied application of thermosensitive polyphosphazenes is injectable hydrogels [62,63,64,65,66,67,68,69]. Figure 17 shows the structure of a thermosensitive polyphosphazene that has been most comprehensively investigated for its use as an injectable hydrogel and tested in a wide range of therapeutic goals. The ratio of the isoleucine ethyl ester groups (a in Figure 17A) to oligoPEG substituents (b) can be used to tune the thermoresponsive behavior, whilst c groups are used for adding functionality, for example drug conjugation or dual cross-linking systems [70,71].

**Figure 18:**
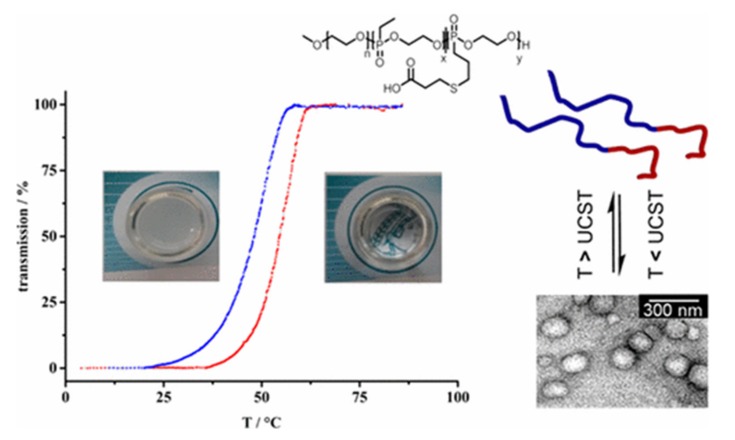
Well-defined poly(phosphonate) block copolymers with adjustable upper critical phase separation (UCST). Left, the USCT measured as the percentage transmission versus temperature, and right, the self-assembly to nanodimensional aggregates. Reprinted with permission from [76]. Copyright (2017) American Chemical Society.

Varying the ratios of hydrophobic and hydrophilic substituents on the phosphorus atoms is also used to tune the LCST of polyphosphoesters [73] and poly(phosphonates) [74] while controlled ROP polymerization techniques allow access to prepare amphiphilic block copolymers. While these polymers use different monomers to achieve the required amphiphilicity, a number of elegant postmodification routes have also been developed—for example, via the addition of dienophiles to furan functionalized polyphosphoesters [75] or thiol–ene addition to alkenes [76]. Post-polymerization functionalization allows the transition temperatures to be readily tuned from a single precursor polymer [75]. They are used for the preparation of, for example, core–shell nanostructures [77] and reversible polymersomes showing UCST transition (Figure 18) [76].

### 2.5. Main-Chain Hydrolysis and Degradation

Degradability in suitable timeframes and in biological media is an important characteristic for many biomedical applications, and it is especially crucial for the clearance of parenterally delivered therapeutics [78]. Thus, for the design of novel polymer-based carriers proposed for parenteral drug delivery, degradation and clearance in a suitable timeframe is essential in order to have a chance of future clinical transition. In certain circumstances, the incorporation of phosphorus in the main chain can infer degradability into the chain due to the propensity of the phosphorus center toward hydrolysis. The direct chemical surrounding of the phosphorus atom determines the rate of degradation of main-chain phosphorus polymers. The strength of the heteroatom bond and the accessibility of the P for H_2_O molecules determine the rate of hydrolysis, which in turn is primarily determined by the steric bulk and hydrophobicity of the organic substituents. This simple relationship allows fine-tuning of the degradation profile, depending on the rate required by the application and the direct environment in which it is to be exposed.

**Figure 19:**
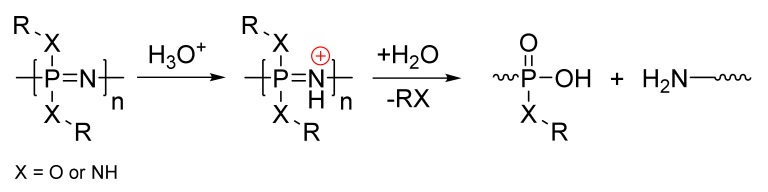
Hydrolytic degradation mechanism of PPz.

Polyphosphazene degradation occurs via the hydrolytic cleavage of substituents to form instable hydroxyphosphazenes that undergo rapid main-chain cleavage to eventually evolve phosphates and ammoniums salts (Figure 19) [79]. The basic main-chain nitrogen atoms tend to become protonated in acidic media [80], accelerating hydrolysis, whilst more basic conditions tend to slow hydrolysis [81].

**Figure 20:**
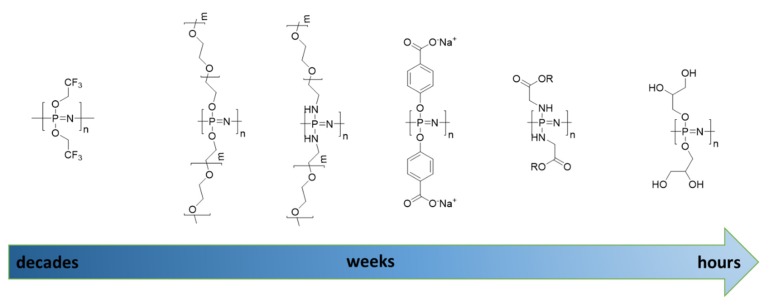
Approximate trend demonstrating how the change of organic substituents on the main-chain phosphorus affects the reported PPz degradation rates in aqueous media.

Figure 20 shows some examples of this. For further details, the reader is referred to recent literature [79].

**Figure 21:**
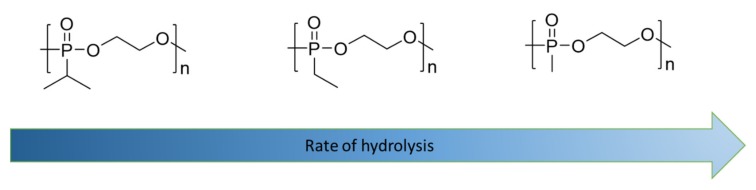
Approximate trend demonstrating how a change of organic substituents on the main-chain phosphorus affects the reported polyphosphonate degradation rates in aqueous media, as reported in [30].

Polyphosphoesters undergo main-chain hydrolysis at rates accelerated in both acid and basic conditions, although in contrast to polyphosphazenes, it is considerably faster in basic compared with acidic media [82]. A simple demonstration of the tunable hydrolysis of main-chain phosphorus polymers was demonstrated by Wurm with a series of polyphosphonates in which an increase in hydrophobic shielding was shown to directly impact the degradation rates (Figure 21) [30].

**Figure 22:**
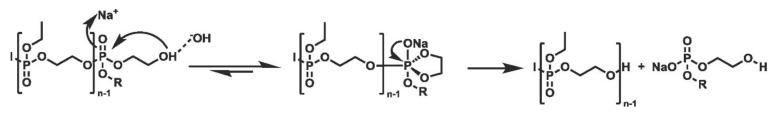
Proposed mechanism for the degradation of PPEs via back-biting from the hydroxyl chain ends. Reproduced from [82], with permission from Elsevier.

The mechanism of degradation for polyphosphoesters has been shown to be predominantly via back-biting from the hydroxyl chain ends (Figure 22) [82]. Hence, influencing these groups can also be effectively used to tailor the degradation rates.

Meanwhile, polyphosphoramidates, having P–N linkages in the main chain, show analogous behavior to polyphosphazenes in that they are shown to be highly labile in acidic conditions [83] but relatively stable in basic conditions, whereby pendant ester hydrolysis occurs rather than main-chain hydrolysis [27]. Thus, a range of polymers with phosphorus in the main chain with different degradation rates toward different environmental conditions can be designed.

### 2.6. Safety and Biocompatibility

In terms of safety and biocompatibility, the broad spectrum of phosphorus-based polymers reported and the generally positive evaluations thereof bode well for the safety of polyphosphoesters and polyphosphazenes. However, it should be clear that the wide variability in chemical structures and functionalization means that sweeping generalizations in terms of biocompatibility are not only unhelpful but incorrect. As with all polymer classes, the discrete polymers and their degradation products must be addressed on an individual basis. Nevertheless, the breadth of studies and positive evaluations, combined with a basic understanding of the expected metabolism products, at least suggest there are no fundamental underlying issues with the use of these polymers. Indeed, trifluoroethyoxypolyphosphazene (marketed as Cobra Pz-F or Polyzene-F) has gained FDA approval as well as progressing to advanced clinical trials in Europe [84,85,86,87], as have microbeads for cancer therapy [84]. While non-degradable, this may help the acceptance generally of this family of polymers in the community.

Biocompatibility is more complex for degradable polymers, as not just the macromolecules must be benign, but also the degradation products and their intermediates. In particular, the organic components used must be carefully considered for these polymer families. For example, the water-soluble and degradable poly[di(sodium carboxylatophenoxy)phosphazene] (PCPP) underwent positive toxicological evaluation by the FDA prior to its use in advanced clinical studies, in which it also showed a good safety profile [88,89]. Furthermore, the degradation products hydroxybenzoic acid, phosphate, and ammoniums salts are benign, with the organic component being a metabolite of n-propyl ester of p-hydroxybenzoic acid and recognized as GRAS (generally accepted as safe) for parenteral applications. Furthermore, amino acid ester functionalized polyphophazenes have had highly promising results in this regard [90]. However, many ill-considered polyorganophosphazenes have been proposed in the literature, with for example toxic aromatic amines as degradation products. Here, careful design and consideration is required from the outset. Similarly, for polyphosphoesters proposed as therapeutics, one needs to be aware of the organic component. For example, Wooley et al. have reported concerns over the problems of toxicity of ethylene glycol, which is produced in large amounts from the degradation of some polyphosphoesters [91]. However, Wurm et al. report alkyl (2-hydroxyethyl) hydrogen phosphate to be the main degradation product of the hydrolysis, not ethylene glycol, which is as yet unknown but reported to be likely toxic by the European Chemical Agency. As with polyphosphazenes, the degradation products resulting from the organic components must be carefully considered for future biomedical products.

## 3. Pharmaceutical Applications

Although polymers sometimes can be active pharmaceutical agents (APIs) in their own right, they are most commonly used as a delivery vehicle of therapeutic agents [92]. Indeed, many major pharmaceutical companies now have R&D programs in this area, and there are a growing number of marketed products (e.g., PEGylated proteins, a PEG-aptamer, and oral polymeric sequestrants). While the first polymer–drug conjugates and block copolymer micelle products (as covalent conjugates) have yet to enter routine clinical use [93], this is a rich and blossoming field in which phosphorus-containing polymers can play a significant part, some highlights of which are detailed in the following sections.

### 3.1. Polymer Chemotherapeutics

The conjugation of small-molecule chemotherapeutic drugs to macromolecules is a widely investigated tactic and is well-known to be able to improve blood solubility, increase blood circulation times, and enhance tumor accumulation [1,94]. Furthermore, smart drug delivery systems can be prepared with stimuli responsive-controlled release. The controlled synthesis methods, hydrolytic degradability, and multivalent nature (for drug loading) make macromolecules with phosphorus in the main chain ideal for such carriers.

#### 3.1.1. Water-Soluble Polymer–Drug Conjugates

Despite the decades-long investigation into polymer–drug conjugates, one of the key challenges in the field remains the transfer into the clinic [94]. Although poly(*N*-(2-hydroxypropyl)methacrylamide) (HPMA), one of the most established polymers for such applications, progressed to different clinical phases, its inherent non-degradability and resulting MW limitations hinders its clinical effectiveness [95]. PEG, on the other hand, while already in routine clinical use, is restricted to low molecular weights due to its non-degradability [96].

**Figure 23:**
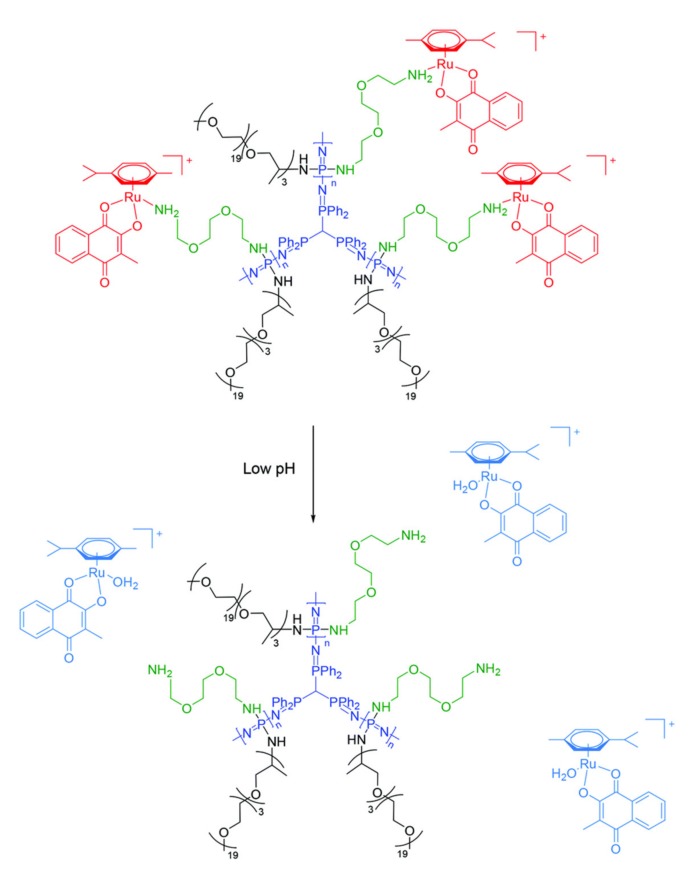
Highly branched molecular structure of polyphosphazene-Ru macromolecular prodrug and its pH-dependent activation. Published by The Royal Society of Chemistry [97].

Several different strategies for polymer therapeutics based on polyphosphazenes have been applied, one of which makes use of the availability of the higher macromolecular architectures described in Section 2.2. A highly branched PPz (Figure 23) has been used as a prodrug for different ruthenium-based metallodrugs [97]. In vivo studies in mice bearing CT-26 colon carcinoma showed that both the solubility and local adverse effects of the free metallodrugs could be improved by conjugation to the polymer. Other approaches, using platinum IV drug derivatives, for example, report increased potency toward cancer cell lines due to their considerably improved cellular uptake; hence, they could help overcome acquired resistance toward the free drug [98]. Furthermore, a series of works have described Pt II drugs conjugated to PPz via diacids on the polymer [99,100,101] and have consistently shown the conjugates to increase the efficacy compared to free oxaliplatin. One recent example using an acid-labile *cis*-aconitic acid linker between the Pt and the PPz showed remarkably better tumor efficacy compared with oxaliplatin at the higher tolerated dose, with lower systemic toxicity [102].

Polyphosphoesters have also been used for drug conjugates of metallodrugs, for example carrying a highly potent dinuclear platinum complex against cisplatin-sensitive as well as cisplatin-resistant cell lines. The PPE functions to decrease its toxicity through the prodrug form and to protect it from binding and subsequent degradation by human plasma protein [103]. Other classical chemotherapeutics conjugated to polyphosphoesters include doxorubicin [104] and chlorambucil [105]. Doxorubicin coupled to PEGylated polyphosphoester, despite poor toxicity in cell culture tests, showed increased tumor growth inhibition compared to the free drug at the same dosage on account of the enhanced permeation and retention (EPR) effect and prolonged blood circulation [104]. Furthermore, hyperbranched polyphosphoester-linked chlorambucil was found to exhibit significant activity against breast cancer cell lines, which is due in part to its ease of cell internalization [105]. The abundance of (terminal) functional groups due to the hyperbranched structure opens up ways toward targeting or theranostic approaches.

**Figure 24:**
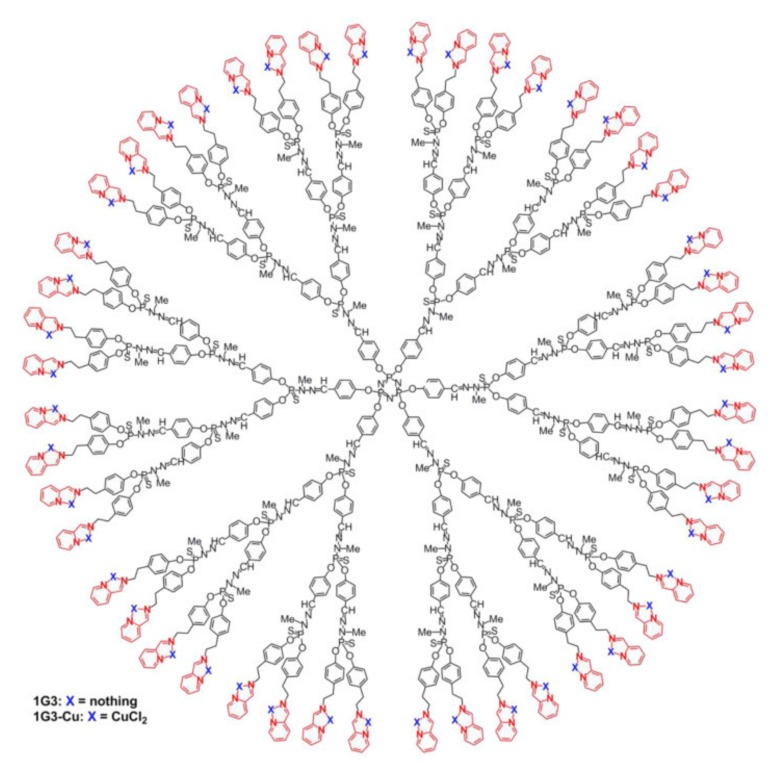
Two-dimensional structure of third-generation dendrimers bearing chelating amine end groups complexing Cu(II). Reproduced from [112], with permission from Elsevier.

Phosphorus dendrimers, bearing phosphorus at every branching point, have been established in a variety of biomedical applications [47]. As shown in the extensive work by Caminade et al., these compounds can act as drug delivery agents [106,107], as well as APIs themselves in a variety of ways [108,109,110,111]. For example, the generation four cationic phosphorus dendrimers can be employed for the delivery of a multitude of compounds including cisplatin, significantly increasing its cytotoxic effectivity [106]. Nevertheless, the dendrimers themselves bear a certain toxicity toward healthy cells for different cell lines [106]. On the contrary, simple dendrimers capped after third generation with *N*-(pyridin-2-ylmethylene)ethanamine complexing Cu(II) (see Figure 24) exhibit a multifold increase in IC50, whereas, for multiple cancer cell lines, the copper complexed compound showed mostly increased antiproliferative inhibition, rendering it a promising antitumor substance [108]. Moreover, acting through a particular mode of action demonstrating proapoptotic activation, these dendrimers open up a new class of antiproliverative compounds [112]. For more details on the topic of phosphorus dendrimers, the reader is referred to the following recent reviews [47,49,113].

#### 3.1.2. Self-Assembling Nanoparticles: Micelles and Polymersomes

Larger, more complex self-assembled nanostructures comprise another vast field of drug delivery systems [114,115]. Both polyphosphazenes as well as polyphosphoesters allow the formation of micelles and polymersomes suitable for such uses, as described above in Section 2.4.

**Figure 25:**
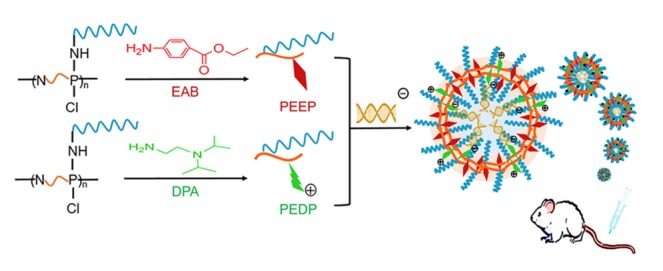
Conceptual pathway toward composite phosphazene polymersomes for miR-200c delivery via physical encapsulation and ionic interaction and subsequent pH-stimulated release in cancer cells upon protonation. Adapted from [116], with permission from Elsevier.

Polyphosphazene polymersomes can be tuned toward coordinated drug-carrier entities, as described by Qiu et al. [54]. For example, for the delivery of RNA (Figure 25), miR-200c, a member of the microRNA-200 family and a remarkably efficient cancer inhibiting substance, still has yet to make an impact due to opposing requirements on delivery. On the one hand, gene transfection efficiency is increased by higher positive charges on the macromolecular carrier; however, these charges also induce stronger systemic toxicity. Combining two amphiphilic PEGylated polyphosphazenes, with ethyl *p*-aminobenzoate (EAB) and *N*,*N*’-diisopropylethylenediamine (DPA) as second substituents, respectively, a composite polymersome both physically encapsulating and ionically interacting with miR-200c has been designed [116]. Additionally, due to the interaction of the protonated DPA group with the negatively charged RNA entity, a nearly neutral nanoparticle is achieved, minimizing systemic toxicity [116].

**Figure 26:**
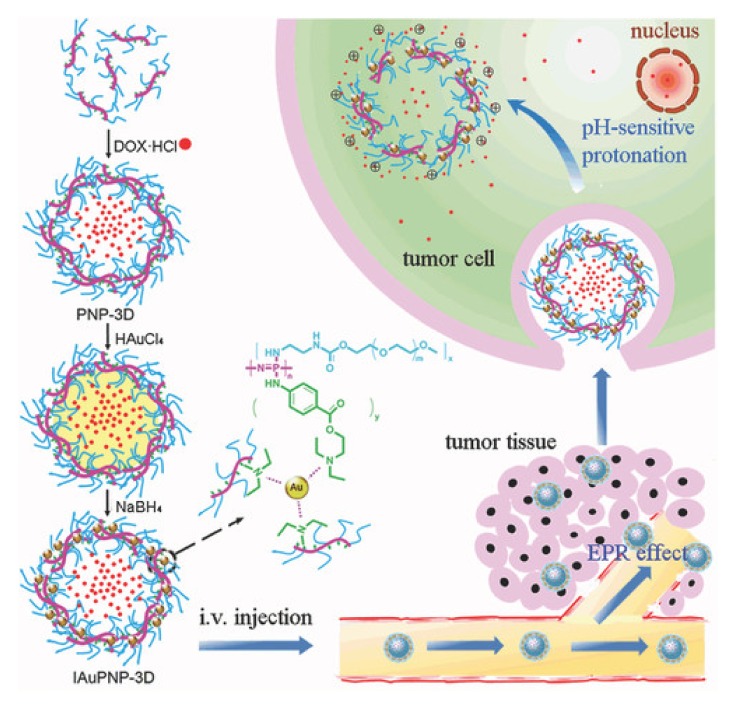
Schematic of polyphosphazene nanoparticles loaded with doxorubicin and gold nanoparticle cross-linked vesicle lamella and their intracellular release upon pH stimuli. Reproduced from [117], with permission from John Wiley and Sons.

While polymersomes are promising carriers of water-soluble therapeutics, a problem for such carriers is the undesired drug leakage during blood circulation, thwarting therapeutic efficiency and increasing side effects. One way to overcome this drawback includes the chemical stabilization of the dynamic structure, for example using gold nanoparticles as inorganic cross-linkers, as shown in Figure 26. This approach effectively increases the compactness of the polymersome toward leakage and allows subsequent controlled release via implemented pH-responsiveness [117].

The incorporation of targeting entities is also applicable for such polymersomes. One approach reports the formation of hybrid polymeric micelles based on poly(bis(carboxyphenoxy)phosphazene) and poly(diallyldimethylammonium chloride), which are covalently and ionically decorated with cholic acid, respectively [118]. Such vesicles loaded with paclitaxel show cytotoxic activity and incorporate drug targeting via cholic acid interactions with the farnesoid-X receptor.

Self-assembled polyphosphoesters are also reported for efficient physical drug encapsulation. For example, Wurm et al. describe a unique system for polymer synthesis based on acyclic diene metathesis [119]. Polyphosphoesters, bridged by distinct numbers of methylene groups between the phosphates, form nanoparticles that are able to encapsulate paclitaxel [120]. The cell toxicity of these particles was shown in vitro, albeit only comparable with the free drug at high drug loading for the same paclitaxel concentration, due to the proposed facilitated release from highly loaded particles. What makes these compounds profoundly interesting for further in vivo experiments is their intrinsic targeting capabilities toward bone tissue due to the interaction of the phosphate groups of the polymer with calcium–phosphate cement [120].

**Figure 27:**
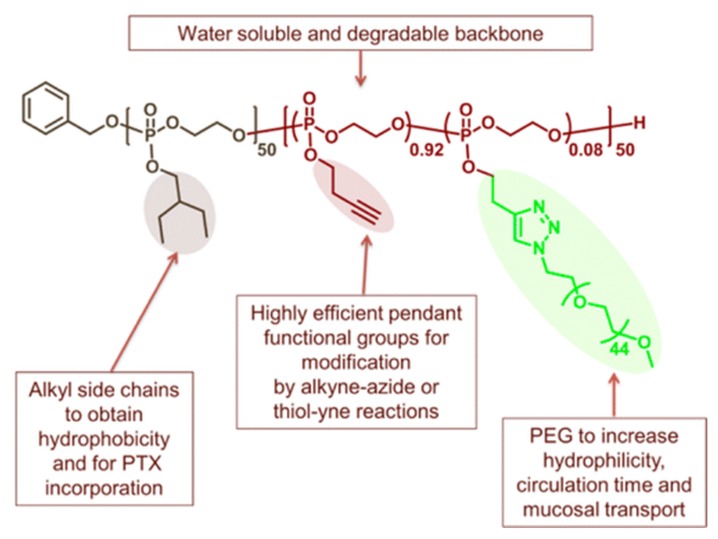
Amphiphilic block terpolymer for drug delivery applications. Reprinted with permission from [53]. Copyright (2015) American Chemical Society.

Cross-linking of either the core or shell of nanoparticles is used to reduce the aforementioned drawback of drug leakage from otherwise dynamic self-assembled formulations. Micelles of a poly(ethylene oxide)-b-polyphosphoester block copolymer bearing unsaturated side groups on the phosphate have been prepared, allowing for the subsequent cross-linking of the hydrophobic core upon UV irradiation [121], thus maintaining the vesicle stability in good solvents and high dilutions. Drug loading via an impregnation process after cross-linking, avoiding any degradation of the active reagent, results in higher loading capacity with a retained release profile compared to non-cross-linked particles formed by nanoprecipitation [121]. In another example, ring-opening polymerization of 2-ethylbutoxy phospholane (EBP) and 2-butynyl phospholane (BYP) was used to prepare the block copolymer depicted in Figure 27 [53]. Copper(I)-catalyzed azide alkyne cycloaddition (CuACC) was used to PEGylate the macromolecule, leading to micelle assembly. Then, cross-linking using a dithiol leads to SCKs. Not only do these SCKs exhibit considerably prolonged drug release half-life times, additionally, the presence of unreacted triple bonds in the shell allow for further possible modification sites and developments toward targeting and theranostic compounds [53].

**Figure 28:**
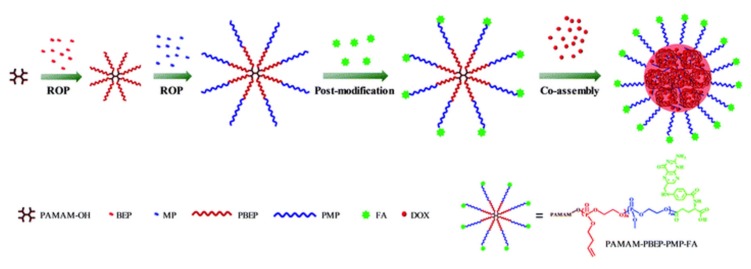
Schematic synthesis pathway of PAMAM–PBEP–PMP–FA multi-arm block co-polymer and subsequent self-assembly into supramolecular micelles. Adapted from [122] with permission from The Royal Society of Chemistry. PAMAM: poly(amido amine), PBEP: polybutadiene-*block*-poly(ethyl ethylene phosphate), PMP: poly(2-methoxy phospholane), FA: folic acid.

Finally, a noteworthy combination of different architectural approaches unites a dendrimer-like secondary structure with self-assembly into micellar tertiary structures [122]. A trivalent block copolymer consisting of poly(amido amine) (PAMAM) as the dendritic core followed by segments of hydrophobic poly(2-butenyl phospholane) (PBEP) and hydrophilic poly(2-methoxy phospholane) (PMP), synthesized via ROP, results in a dendrimer-like structure, as shown in Figure 28. Employing a low-generation PAMAM core and further modifying it by polyphosphoesters not only avoids complicated synthesis but also compensates for the lower drug-loading capacity. The drug-loading capacity is further enhanced by the self-assembly of the amphiphilic structure into micelles, as shown in Figure 28, which itself possesses the advantage of reinforced stability against dissociation due to stronger physical interactions of the multi-arm polymers. These supramolecular carriers allow for a multi-step drug release-minimizing primary leakage in the bloodstream affected by the diluted environment. When capped with folic acid (FA) as a targeting agent, they demonstrate high tumor suppression alongside low body toxicity [122].

#### 3.1.3. Injectable Hydrogels

Adverse effects due to the systemic toxicity of chemotherapeutics pose one of the major obstacles of current cancer treatments. Injectable hydrogels aim to overcome this by localizing drug release. They circumvent systemic delivery, provide high concentration of the drug at the tumor site, and furthermore enable a simple route toward the simultaneous delivery of synergistic or multiple drugs [123,124,125,126]. An extensive portfolio of various examples of injectable hydrogels based on polyphosphazenes can be found within the publications of Song et al., from which just a few recent reports highlighting the advantages of hydrogels are described here in more detail. Doxorubicin and paclitaxel both display their cytotoxic abilities in a cell-cycle specific manner, making the co-administration of these compounds in a synergistic way tedious and clinically inconvenient. Moreover, simultaneous administration has possible antagonistic effects. Tuning the properties of a PPz hydrogel considering the difference in the hydrophilicity of these drugs enables a time-resolved, continued release, leading to a synergistic effect [127]. Such formulations showed excellent results concerning toxicity, reporting no death subjects over the course of the experiment in contrast to the free drug in solution. Nevertheless, problems regarding the tumor relapse of certain tumor models require further investigation alongside the tricky application via intratumoral injection [127].

**Figure 29:**
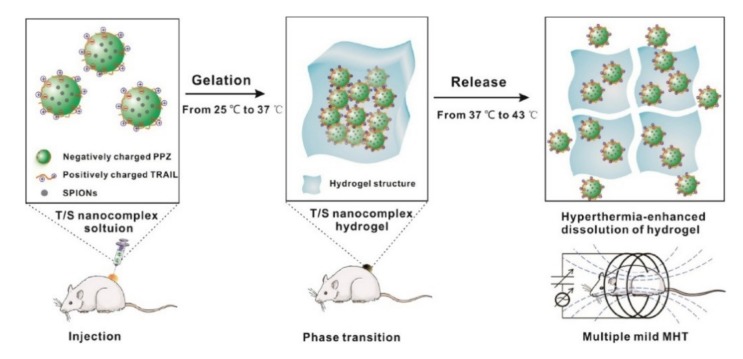
Schematic path of administration and mode of action of tumor necrosis factor-related apoptosis-inducing ligand (TRAIL)/superparamagnetic iron oxide nanoparticles (SPION) loaded injectable hydrogels. Reproduced from [65], with permission from Elsevier.

Another interesting example includes the usage of tumor necrosis factor-related apoptosis-inducing ligand (TRAIL), as depicted in Figure 29 [65]. TRAIL selectively induces apoptosis in cancer cells, sparing healthy ones, yet due to the short biological half-life and resistance of some tumors, it was not able to unfold its potential. Taking advantage of the versatility of substituents for polyphosphazenes, a suitable hydrogel for TRAIL release can be realized that undergoes in vivo gelation upon injection. Magnetic hyperthermia therapy (MHT) as an adjuvant medical treatment via incorporated superparamagnetic iron oxide nanoparticles (SPIONs) further enhances effectivity, acts as release stimuli, and even restores sensitivity in inherently TRAIL-resistant cancer cells. The SPIONs additionally allow for a long-term monitoring of the therapeutic outcome via MRI (magnetic resonance imaging) in a theranostic approach [65].

**Figure 30:**
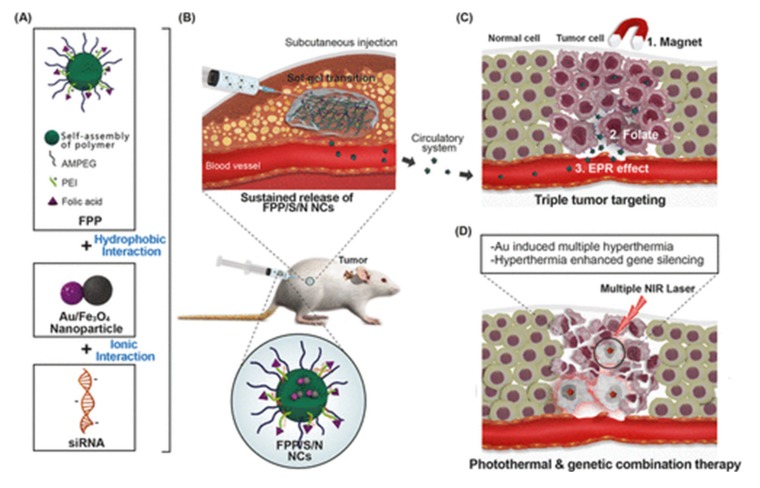
Nanocapsules (NC) hydrogel administration by single SC injection for long-term gene/photothermal combination therapy. (**A**) NC assembly of PPz(FPP)/siRNA/NP. (**B**) Temperature-responsive sol–gel phase shift of injected NCs into hydrogels and subsequent sustained release. (**C**) Systemic delivery of the NCs and triple-targeting via EPR/folate/magnetism. (**D**) Combination therapy of siRNA and hypothermia leads to tumor death. Reprinted with permission from [128]. Copyright (2019) American Chemical Society.

Both the aforementioned examples have in common that their application takes place via intratumoral injection, therefore sharing the drawback of accessibility of the injection site. The approach of a subcutaneous (SC)injection of polyphosphazene-based nanocapsules (NCs) undergoing a sol–gel phase shift at body temperature, Figure 30, bypasses this forming a drug-release depot. Released NCs loaded with siRNA as an active agent, as well as Au−Fe_3_O_4_ nanoparticles for synergistic hypothermial treatment, have a threefold mechanism to enhance tumor accumulation, namely via the EPR effect, folate conjugation, and magnetic interaction. Compared to simple IV injection, this administration pathway significantly increases tumor accumulation compared to other organs [128].

**Figure 31:**
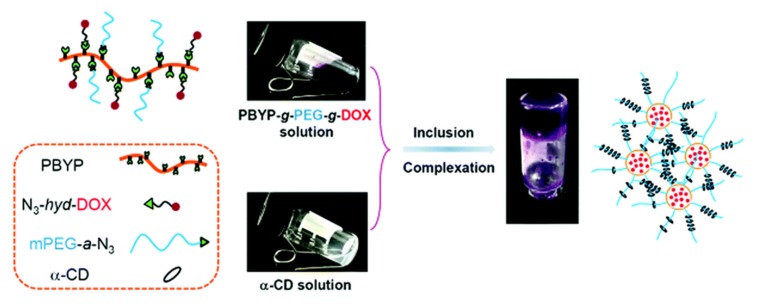
Injectable hydrogel formed upon the inclusion complexation of PEG side groups grafted to a polyphosphoester backbone modified as a polymeric prodrug for doxorubicin. Reproduced from [130] with permission from The Royal Society of Chemistry.

Polyphosphoester-based injectable hydrogels have not been investigated to the same extent as PPz derivates. Nevertheless, applying different cross-linking methods, some early results toward drug delivery are reported employing ionic interaction [129], inclusion complexation [130], or covalent bond formation alongside ionic interactions [131]. Poly(propylene phosphate) in combination with Ca^2+^-ions undergoes a sol–gel transition, forming ionic hydrogels. Matching polymer content and ion concentration, the transition temperature of the gelation can be tuned allowing for an administration as injectable hydrogels for the delivery of plasmid DNA [129]. Another stimuli responsive network, cross-linked by the inclusion complexation of grafted PEG and α-dextrin (Figure 31), was tested for the pH-dependent release of doxorubicin. The system, based on CuAAC-modified poly(butynyl phospholane), bears acid labile acetal and hydrazone linkers and shows a near 10-fold drug release at pH 5 compared to pH 7.4 over the same time span. In addition to the covalently linked doxorubicin, these hydrogels may also be loaded physically, enabling a combination therapy approach [130]. Free radical polymerization of a novel phosphotriester cross-linker followed by elimination reaction of the prepolymer results in unique materials with high phosphodiester content [131]. These ionic hydrogels, in addition to excellent cell and hemocompatibility, show charge-governed release of fluorophore model compounds through interaction of the anionic matrix with the loaded compound positioning these innovative materials as potent candidates for in vivo investigations [131].

#### 3.1.4. Photodynamic Therapy

**Figure 32:**
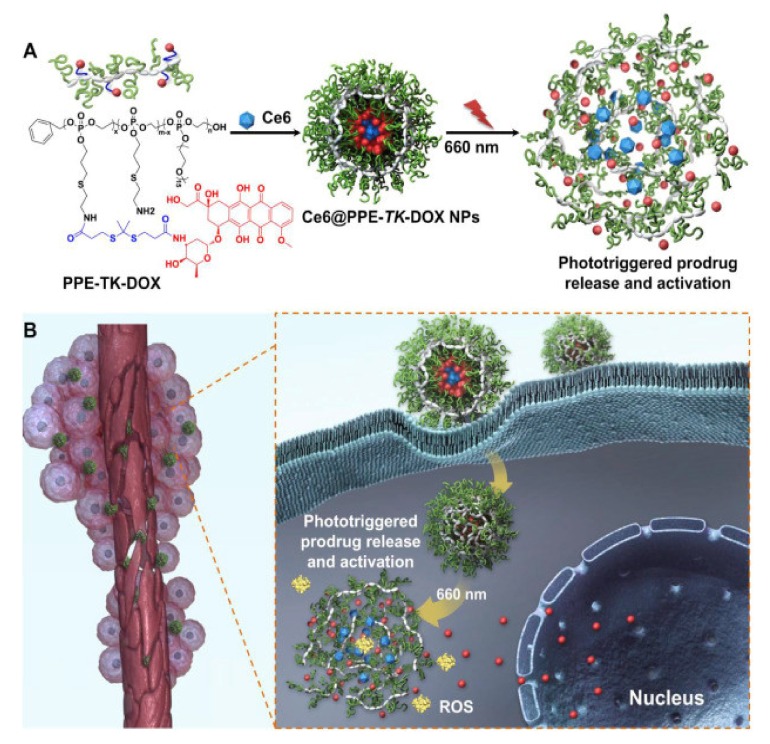
Schematic of photo-stimuli responsive polyphosphoester nanoparticles. (**A**) Structure of polyphosphoester-conjugated doxorubicin using a reducible thioketal linker and its co-self-coassembly with Ce6 to photoreactive nanoparticles. (**B**) Light irradiation of the nanoparticles at the tumor site results in localized reactive oxygen species (ROS) formation and subsequent cleavage of the reducible linker, releasing active doxorubicin. Reproduced from [138], with permission from Elsevier.

Macromolecules can also be used in targeted photodynamic therapy (PDT) [132] to enact light-induced activation such as the generation of reactive oxygen species (ROS) and/or release of drugs upon irradiation [133]. In this context, PPz and PPE, while themselves photochemically inert, have been investigated as carriers for PDT. For example, the notoriously poor aqueous solubility of hypericin, a naturally occurring clinical photosensitizer, could be enhanced significantly through conjugation with water-soluble PPz [134]. The PPz–hypericin conjugates were shown to be efficient inducers of cell apoptosis upon irradiation at 610 nm [135]. PPz loaded with photocleavable coumarin moieties have also been developed, which, due to their photochemically induced degradation with light in the visible region, have potential in the photochemical release of drugs [136]. Meanwhile, the photosensitizer chlorin e6 (Ce6) has been loaded into acetal-linked hyperbranched polyphosphoesters [137]. The pH-triggered release, triggered by acetal cleavage upon endocytosis, was demonstrated to enhance the intracellular ROS generation [137]. Furthermore, a copolymer of a PEGylated and an allyl-bearing phosphoester-monomer is further adapted by post-polymerization modification reactions resulting in the polymer depicted in Figure 32A, bearing doxorubicin linked via reductively labile thioketals. This polymer self-assembles into micelles and encapsulates Ce6 in its hydrophobic core. Upon circulation of the nanoparticle to the tumor site, a precise irradiation with red light leads to the formation of ROS by the photosensitizer Ce6 and results in release of the doxorubicin (Figure 32B) [138].

### 3.2. Protein PEGylation

**Figure 33:**
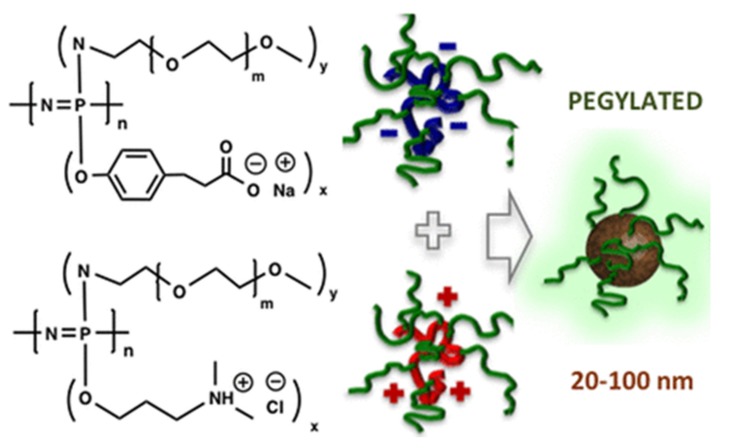
Degradable cationic and anionic PEGylated PPz spontaneously assemble into nanoparticles for noncovalent PEGylation of proteins in vivo. Adapted with permission from [142]. Copyright (2018) American Chemical Society.

Polyethyleneglycol (PEG) is well-known as a “stealth” molecule protecting conjugated therapeutics from phagocytosis and the resulting removal from the bloodstream, as well as protecting against enzymatic degradation [139]. Not only regarded as the “gold standard” of water-soluble biomaterials, it is also generally regarded as safe by the FDA and practically the sole defined polymer platform for biomedical applications used clinically outside research laboratories [140,141]. Nevertheless, in recent years, several reports stating the important limitations of PEG, such as its non-degradability and accumulation in vivo as well as PEG immunogenicity have emerged, calling for alternatives [140]. To achieve high molecular weights with short PEG chains, hybrids of PPz with PEG and PEG-type oligomers can be prepared; for example, poly(ethylene oxide-co-propylene oxide) linked via amino acids to PPz results in water-soluble polymers with tunable degradability [80]. Furthermore, mixed macrosubstitution allows the synthesis of PEGylated cationic and anionic PPz, bearing carboxylic acid and tertiary amine functionalities, respectively [142]. A mixture of both leads to the formation of polyelectrolyte complex micelles that are capable of protein encapsulation, as schematically depicted in Figure 33. Noncovalent modification has shown to successfully reduce the antigenicity of encapsulated L-asparaginase in in vitro tests and further enhance thermal and proteolytic stability. In contrast to conventional covalent PEGylation, this non-covalent concept may overcome the possible blockage of active sites of the protein, which is known to occur for covalently bound PEG chains [142].

**Figure 34:**
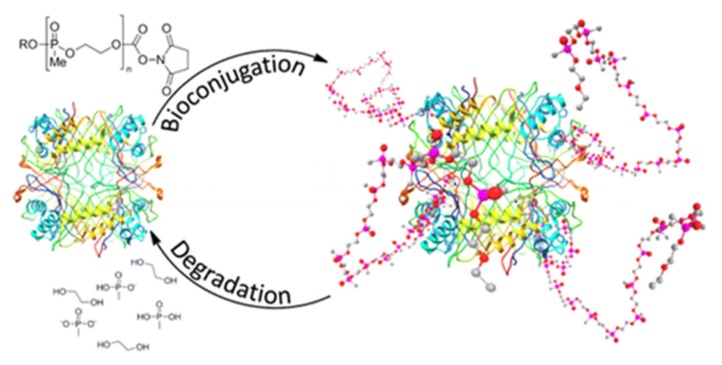
PPEylation; covalent conjugation of degradable poly(phosphoesters) to proteins. Reproduced from [143] (https://pubs.acs.org/doi/abs/10.1021/acs.biomac.6b01107), with permission from the American Chemical Society.

Wurm et al. coined the term “PPEylation” for the replacement of PEG with degradable polyphosphoesters for bioconjugation, as depicted in Figure 34. Combinations of poly(ethylene methylphosphonate) (PMeEP) and poly(ethyl ethylene phosphate) (PEEP) with bovine serum albumin (BSA) and uricase (UC) [143] and BSA, catalase, and myoglobin [144,145] respectively, were investigated and show comparable results to PEGylation. ω-Functionalization of the PPE with succinimidyl carbonate enables well-known coupling to ε-amine residues of lysine. In general, the applicability tested by conjugation to BSA as a model protein shows successful conjugation with different molecular weight polymers and polymer–protein ratios, as well as complete degradability, based on extensive testing via SEC-MALLS, MALDI-ToF, and SDS-PAGE [143,145]. The enzymatic activity of conjugated proteins was further examined based on different assays specific for the respective enzyme and displayed comparable results to PEG in all cases [144,145]. Additionally, a detailed investigation of the interaction and influence of the polymer with regard to relaxation dynamics and conjugate conformation was performed, giving insight into their relation and biophysical characteristics [146,147,148].

### 3.3. Smart Endosomal Release

**Figure 35:**
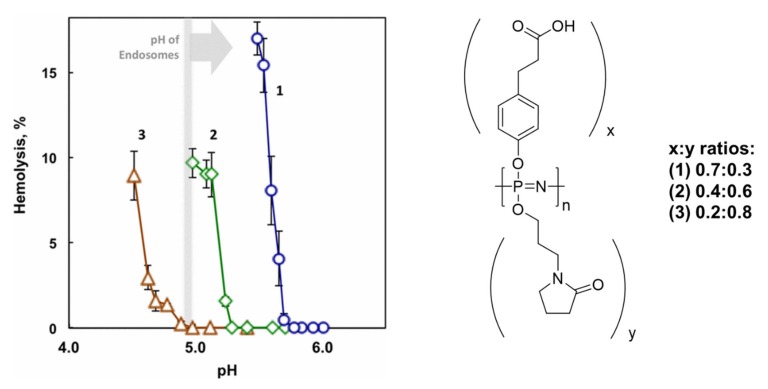
The membrane disruptive activity of multifunctional carriers as demonstrated with hemolysis studies. The onset of hemolytic activity moves to higher pH with increasing n-carboxylic acid side groups in the polymer. Adapted with permission from [150]. Copyright (2017) American Chemical Society.

A critical limiting factor in pharmaceutical efficacy is their intracellular trafficking [149]. Macromolecules are generally compartmentalized by cells in endosomes; hence, this process is critical in determining the rate and extent of uptake of conjugates. In particular for the delivery of biomacromolecules and proteins, the escape from this compartment into the cytosol is important for the overall efficacy. To this end, important work by Andrianov and coworkers has demonstrated that polyphosphazenes substituted with aliphatic carboxylic acid groups, while showing no membrane-disruptive properties at neutral pH values, become protonated in the endosomal pH range (Figure 35). The more hydrophobic nature of the protonated PPz leads to membrane disruption, resulting in cytosolic release of the payload [150]. PCPP–PEO complexation has also been demonstrated to have a pH-dependent membrane disruptive activity [151]. This smart endosomal release effect has recently been utilized for gene delivery, a therapy for which endosomal survival and release of the oligonucleotide is decisive [152]. While a number of researchers have used tertiary and primary amine-functionalized PPzs as cationic carriers for genes [153,154,155], in this work, the combination of carboxylic acid groups with amines was used to prepare ideal carriers for both pDNA and siRNA delivery. The conjugates reduced cell renewal in vitro, and it was shown in vivo that siRNA can silence DYRK1A, which is a gene implicated in glioblastoma delaying tumor growth [152]. This is presumed to be due to the increased endosomal release. While showing comparable gene delivery efficacy to commercial standards (PEI or lipofectamine), the biodegradability and lower cytotoxicity make them excellent candidates as carriers for gene delivery [152].

### 3.4. Immunology

#### 3.4.1. Cancer Immunotherapy

**Figure 36:**
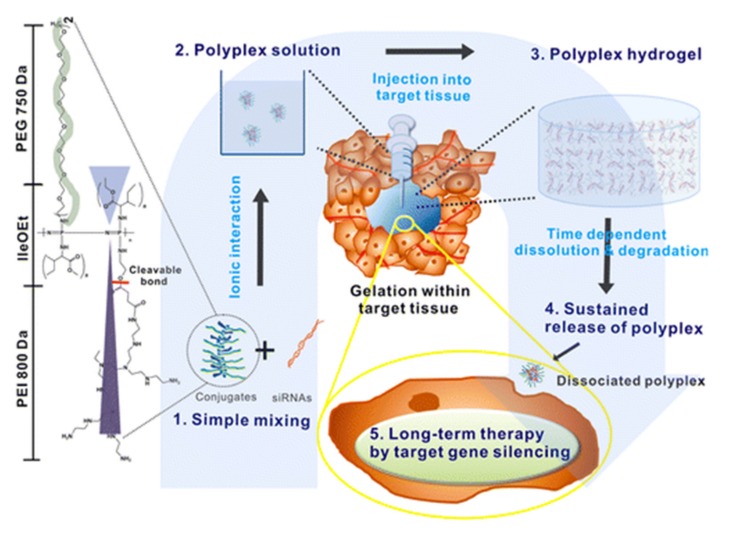
Scheme of polyethyleneimine (PEI)–siRNA polyplex hydrogels allowing localized long-term gene delivery. PPz-conjugated PEI and siRNA polyplex formed via ionic interaction undergo sol–gel transformation at body temperature in the target tissue. The dissolution and degradation of the hydrogels release the polyplex in a time-dependent manner, achieving long-term therapy gene silencing. Reprinted with permission from [72]. Copyright (2012) American Chemical Society.

Immuno-oncology, in contrast to more conventional chemotherapy, acts through a unique mechanism of action aiming to promote the body’s own immune response toward the malignant cells, encompassing the advantage of less serious side effects, among others [156]. Gene delivery, one branch of immunotherapy, relies on the transport of intact nucleic acids into the cell, efficient transfection, and subsequent protein expression. Polymer-based systems are particularly promising for such applications [157]. For example, polyplexes formed by PPz conjugated polyethyleneimine (PEI) and siRNA, have been shown to undergo sol–gel phase transitions at body temperature, forming a thermosensitive injectable hydrogel (Figure 36) [59]. Following the dissolution and degradation of the injected gel, a local sustained release of the polyplex and efficient transfection of cells can be observed. A long-term gene silencing effect ensues, resulting in tumor growth inhibition observable 30 days after a single injection [72]. Polyphosphazene modified with *N*,*N*’-diisopropylethylenediamine (DPA) and monomethoxy poly(ethylene glycol) (mPEG) as hydrophobic and hydrophilic groups, respectively, can self-assemble into polymersomes [158]. Encapsulated IL-12 plasmids can be successfully delivered without enzymatic degradation of the DNA, as well as preventing agglomeration with BSA and subsequent plasma clearance, which is one of the major drawbacks of classical cationic delivery vesicles [158]. Similarly, a block-copolymer of mPEG, branched polyethyleneimine (bPEI), and poly(2-ethylbutyl phospholane) (PEBP) can be utilized for the transport of chimeric antigen receptor (CAR)-encoding plasmids. The high density of cationic charges on the bPEI allows ionic interaction with the phosphate DNA backbone; however, in combination with PEG and PEBP, these charges are enveloped in a micelle, decreasing the cytotoxic effect [159].

**Figure 37:**
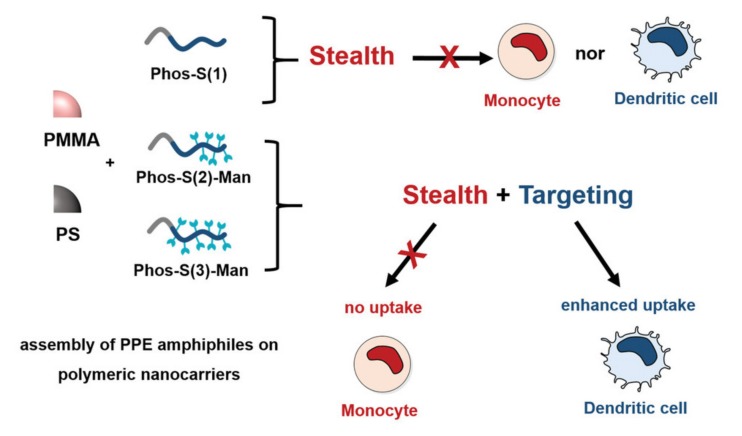
Mannose-conjugated PPE adsorb to polymeric nanocarriers conferring stealth and targeting abilities. Adapted from [164], provided under CC BY 4.0 license.

T-cell activation is another target of immunotherapy, which is mainly achieved indirectly by activating dendritic cells (DCs) to present tumor-associated antigens [160]. Acting as a macromolecular prodrug, polyphosphazenes bearing imidazoquinolines targeting Toll-like receptors (TLR) can be used for the delivery of adjuvants, boosting the DC activation [52]. On top of the advantage of direct transport by the prodrug via endocytosis to the TLR-7 and -8 receptors, which are located in the endosome, the macromolecules further exhibit specific release due to attachment via linkers susceptible to endosomal pH values. Furthermore, “stealth” properties can be simply adapted to pre-synthesized nanoparticles by PPE adsorption, as reported by Wurm et al. for targeted DC delivery [161,162,163]. Model nanoparticles of PMMA and PS coated with the PPE show low binding affinity toward dendritic cells but can target the latter when a mannosylated coating is used, as shown in Figure 37. Furthermore, the targeting persists, even after the incubation of the particles in human blood plasma and the formation of a protein corona, effectively activating receptor-mediated uptake [164].

**Figure 38:**
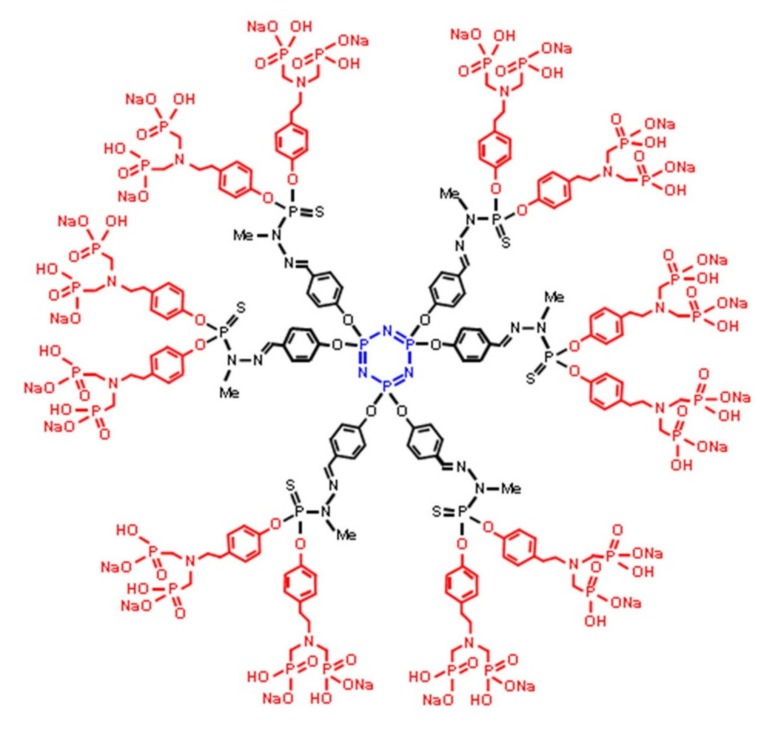
2D structure of the monosodium salt of amino-bis(methylene phosphonate)-capped poly(phosphorhydrazone) dendrimers. Adapted from [165], with permission from Elsevier.

Phosphorus dendrimers have also been applied for immunotherapy, albeit in a rather unique, indirect manner. Natural killer cells, as part of the innate immunity are potential accomplices against malignancies and can be proliferated ex vivo before re-injection [165,166]. The addition of monosodium salts of amino-bis(methylene phosphonate) capped poly(phosphorhydrazone) G1 dendrimers, as depicted in Figure 38, to in vitro cultures of peripheral blood mononuclear cells (PBMC) from healthy volunteers showed a 16-fold increase in the total number of NKs compared to threefold without dendrimers over the course of two weeks. Additionally, the dendrimers selectively promote the proliferation of NK cells shown by a steep increase in percentage among PBMC in contrast to the control. Finally, in vivo tests in a xenograft murine model demonstrated the cytotoxic effect of the amplified NK cells, promoting the dendrimers as effective adjuvants for ex vivo proliferation [165].

#### 3.4.2. Vaccine Adjuvants

**Figure 39:**
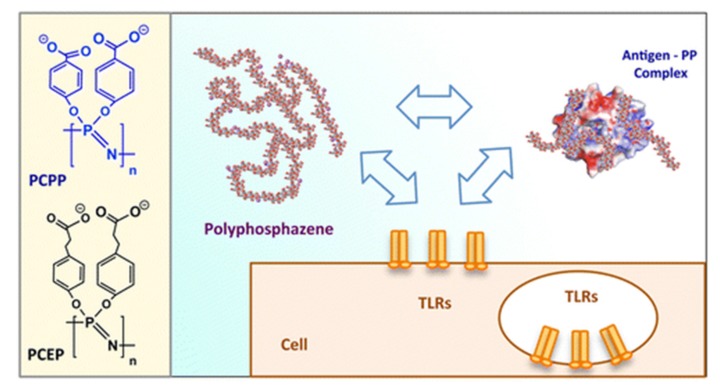
Chemical structures of some polyacid poly(organo)phosphazenes. PCEP: Poly di(sodium carboxylatoethylphenoxy)phosphazenes, capable of an immune activation, thought to be via activation of Toll-like receptors (TLRs), as well as forming conjugates for the delivery of vaccine antigens. Reprinted with permission from [172]. Copyright (2016) American Chemical Society.

One of the most advanced and promising therapeutic applications for polyphosphazenes are their use in vaccines. Ionic polyphosphazenes, pioneered by Andrianov [167,168,169,170,171], have been found to be outstanding vaccine adjuvants, activating innate immune responses by the activation of TLRs as well as serving to transport, protect, and stabilize the loaded antigens, whilst being able to present them to immune competent cells [172]. Anionic PPzs are proposed as a replacement for aluminum-based adjuvants, offering improved systems with longer lasting and higher immune responses. The immunoadjuvant activity is also reported to be significantly higher than similar organic ionic polymers such as polyacrylic acid [173]. The exact reasons for this are yet not fully understood [174], but it would appear that the high density of binding sites and high conformational adaptability of the PPz main chain can aid protein binding and presentation [167]. The most investigated structures, PCPP and poly[di(sodium carboxylatoethylphenoxy)phosphazene] (PCEP) (Figure 39), have shown positive results in combination with a diverse range of antigens in large animals [175,176] and phase I and phase II clinical trials in humans [174,177,178], and they have been developed as microneedle patches [179,180] for intradermal administration.

### 3.5. Antimicrobial Polymers

**Figure 40:**
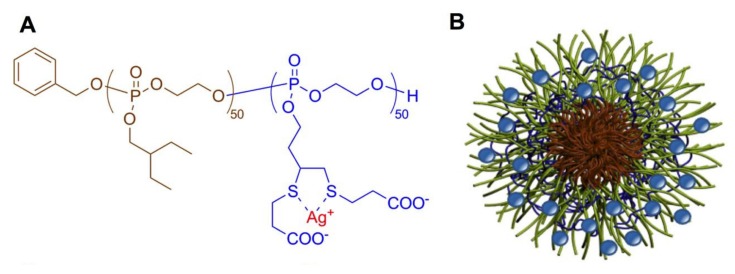
Thioether-functionalized PPE diblock copolymers (**A**) can be loaded with silver and form SCK nanoparticles (**B**) with excellent antimicrobial activity. Adapted from [183].

The combination of rising antibiotic resistance and tricky antimicrobial drug discovery make the preparation of new antimicrobials a pressing societal issue [181]. A series of PPE-based aerosolizable silver-based antimicrobials have been developed and investigated for their use in the treatment of pulmonary infections [182,183]. The SCKs have been labeled with ^111^Ag [184], and it could be shown that the PPE SCK nanoparticles (Figure 40) had good retention in the lungs of healthy mice up to 24 h after aerosol dose administration at considerably higher amounts than in most other organs [183]. The anionic PPEs used in these studies were prepared via thiol–ene post-polymerization functionalization reaction (see Section 2.1.5) with 3-mercaptopropanoic acid to give thioethers, to which the active silver moieties could be bound. A similar post-polymerization functionalization of alkyne-bearing PPEs has also been used to conjugate cysteine-bearing antimicrobial peptides onto PPEs [185], which was demonstrated to enhance the bactericidal activity of the antimicrobial peptide.

### 3.6. Imaging Applications

**Figure 41:**
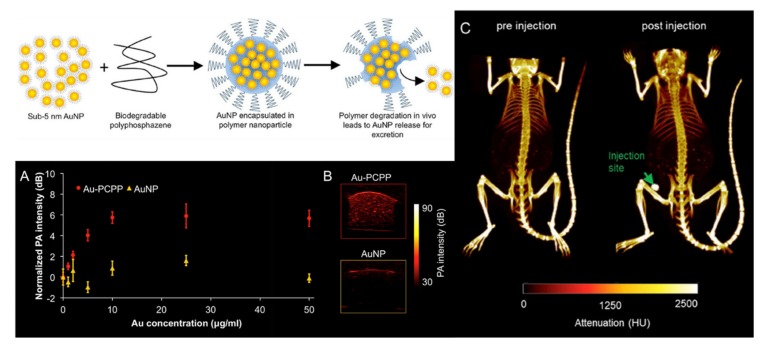
The concept of poly[di(sodium carboxylatophenoxy)phosphazene] (PCPP)-based gold nanoparticles (AuNPs) and therapy use in computed tomography (CT) imaging. (**A**) and (**B**) show the CT contrast enhancement with PCPP-based AuNPs compared to AuNPs. (**C**) Strong CT contrast can be observed upon the intramuscular injection of Au-PCPP in mice. Adapted from [186], with permission from Elsevier.

The multivalent nature of polymers with phosphorus in the main chain enables the dual loading of labels with therapeutic agents, hence facilitating the preparation of theranostics. This has been utilized for polyphosphoesters, for example, for paclitaxel delivery systems designed by Wooley [53], as well as some other examples discussed in Section 3.1.1 and Section 3.1.2. Similarly, the same polyphosphazene-based injectable hydrogels described in Section 3.1.3 have also been used for magnetic resonance imaging, for example through the co-inclusion of superparamagnetic iron oxide nanoparticles (SPIONs) [65]. Meanwhile, Cormode et al. have also successfully applied anionic polyphosphazenes as contrast agents for X-ray computed tomography (CT) and photoacoustic imaging (PA) in combination with gold nanoparticles (AuNPs). Small AuNPs (≈5 nm) were agglomerated into larger stable particles (≈50–100 nm) using PCPP as a binding agent (Figure 41) [186]. The larger particles show longer circulation times and excellent contrast, but degradation of the PCPP leads to disassembly of the particles to give excretable sub-5 nm AuNPs, thus facilitating their safe application. The same tactic has also been extended to multimetal particles containing gold, tantalum, and cerium, which were observed to have higher and more stable CT contrasts than agents made from a single contrast-generating material [187]. Furthermore, oxidation-sensitive polyphosphazenes have been applied in combination with PCPP and investigated for the detection of reactive oxygen species (ROS) [35]. High levels of ROS are commonly associated with inflammations and cancer. Disassembly of the particles leads to a decreased PA signal, but since the CT signal is independent of the direct local environment of the metal, the difference in the signals could be used to detect areas of high ROS.

## 4. Biomaterials

### 4.1. Thromboresistant Coatings

**Figure 42:**
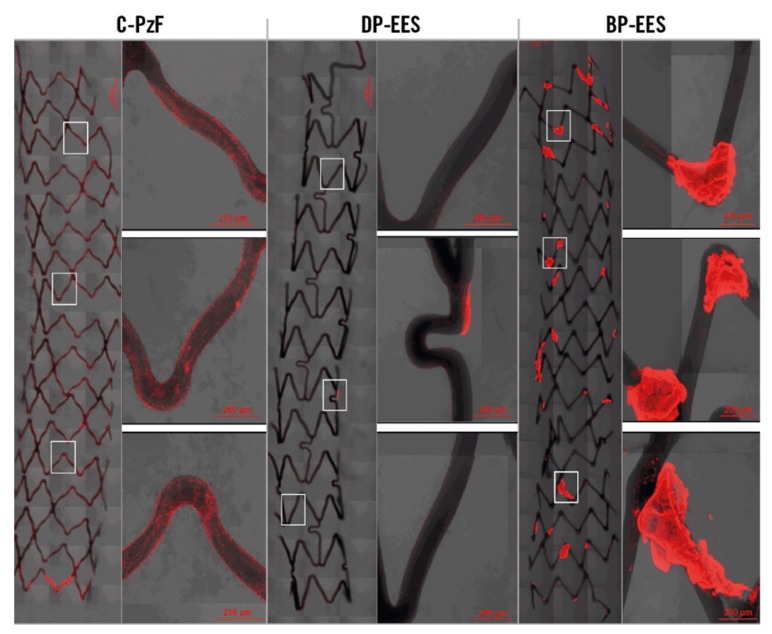
Representative images showing the absence of clumping (which would indicate platelet aggregation) for polyphosphosphazene (C-PzF) coated stents in comparison to other state-of-the-art durable polymer everolimus-eluting stents (DP-EES) and bioabsorbable polymer everolimus-eluting stents (BP-EES). Republished with permission of Europa Group, from [85]; permission conveyed through Copyright Clearance Center, Inc.

Fluorinated polyphosphazenes have been clinically applied and are commercially available for a number of years as bioinert denture linings [188], with their main features being the unique softness in combination with bioinertness. More recently, coatings for stents have also emerged and progressed to advanced clinical trials also using trifluorethoxy polyphosphazene [NP(CF_3_CH_2_O)_2_]_n_-based materials [84], which are sometimes referred to as Cobra-PzF or C-Pz-F (Figure 42). The amassed data suggest both antithrombotic and superior healing characteristics for PPz coated stents, which is thought to be due to its ability to bind albumin, forming a “biomimetic” layer, which results in superior anti-inflammatory and thromboresistant properties compared to uncoated stents and indeed alternative clinical coatings [85]. Co-substitution can also be used to enhance the properties of such polymers with a view to their use in blood-contacting medical devices [189,190], for example water-soluble hemocompatible, sulfo–fluoropolymers [191].

In an alternative approach to antithrombotic effects, degradable amino acid ester-substituted polyphosphazenes have been investigated as NO-releasing coatings via cosubstitution with *S*-nitrosothiols [192,193]. Nitrogen monoxide is known to be a critical signaling molecule and exhibits antithrombotic effects. The polymers were shown to be capable of physiologically relevant NO release for an extended duration, with the high loading and degradability making polyphosphazenes an ideal basis for such biomaterials.

### 4.2. Degradable Scaffolds for Tissue Regeneration

**Figure 43:**
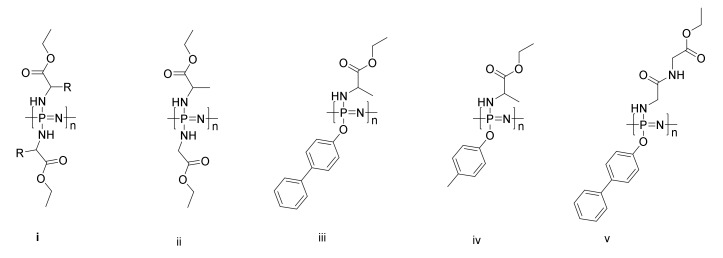
Overview of the chemical structures of some polyphosphazenes proposed as degradable scaffolds.

The use of degradable polymer based scaffolds to assist artificial cell growth toward three-dimensional tissues is a widely used tactic in regenerative medicine and tissue engineering [194], and it has been widely studied for phosphorus-containing polymers [195]. Degradable scaffolds of polyphosphazenes have been heavily investigated in this regard, primarily by Allcock and Laurencin since the early 1990s [196], and indeed, many recent reviews have detailed their progress of this field [197,198,199]. Hence, only a brief overview is given here. The main premise of using polyphosphazenes is their tuneable degradation rates, which can in principle be tailored to match cell growth. The most investigated PPzs are functionalized with amino acid esters (Figure 43i), with the release of amino acids upon hydrolysis helping to boost cell growth [200]. A wide range of cells has been successfully cultivated on amino acid ester-substituted PPz-based matrices [197]. A further important property is that the resulting degradation products, phosphates, and ammonium salts are considerably less acidic than those of the poly(α-ester)s that currently dominate the field [201]. This is an important feature, as the local acidic pH values from hydrolyzing poly(α-ester)s are detrimental to cell growth. Indeed, it has been shown that the release of ammonia and phosphates leads to a buffering action [201], which can even help neutralize the acid released during the degradation of the poly(lactic-co-glycolic acid) (PLAGA) in blends of the two polymers.

While amino acid ester-substituted polyphosphazenes produce polymers with suitable degradation profiles, the mechanical properties are insufficient for some load-bearing applications [202]; hence, co-substitution, for example with phenyl groups, is used to improve the chain stiffness and to increase T_g_ values to above the physiological temperature [203]. A systematic study of such cosubstituted PPzs showed improved mechanical properties but significantly slowed degradation rates for the more hydrophobic substituents. Indeed, while a film of PPz with glycine and alanine substituents (Figure 43ii) showed complete degradation in 7 weeks at 37 °C in PBS (phosphate-buffered saline) buffer, those substituted with (p-phenyl phenoxy) or methyphenoxy (Figure 43iii and iv respectively) showed only minimal mass loss in this timeframe [203]. An alternative approach, pioneered also by the collaboration of Allcock and Laurencin is the blending of PPz with poly(α-ester)s. This approach has recently been reviewed in detail [204]. While the buffering capacity of PPz improves PLAGA-based products, the major challenge is the blend miscibility. Possible ways to overcome this are for example by block copolymers [205] or co-substitution with moieties such as for example, dipeptides, which are capable of hydrogen bonding with PLAGA (Figure 43) [206].

**Figure 44:**
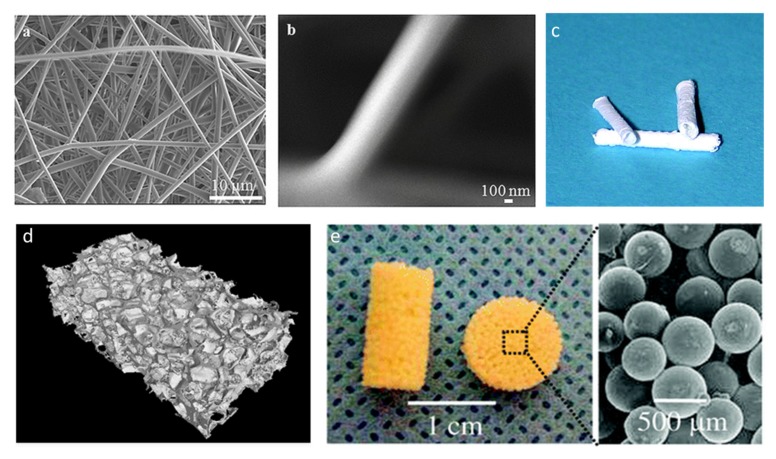
Representative fabricated structures of amino acid ester polyphosphazenes (**a**) poly(lactic acid) PLA-blended fibers, (**b**) an SEM image showing the smoothness of the blended fiber [210]. (**c**) electrospun fibers of ethyl glycine/ethyl alanine PPz [209], (**d**) photochemically polymerized glycine-based PPz [208], and (**e**) microsphere-based monoliths composite microspheres with 100 nm sized hydroxyapatite and phenylalanine ethyl ester-substituted PPz [213]. Adapted (**a**) and (**b**) from [210], (**c**) [209], and (**d**) [208], with permission from John Wiley and Sons. (**e**) Adapted with permission from [213]. Copyright (2008) American Chemical Society.

Aside from blending with poly(α-ester)s, amino acid ester-substituted polyphosphazenes can be processed into 3D matrices by the sintering of bioerodible microspheres [207] or photolithography via pendant vinyl groups [208]. Figure 44 shows some examples of fabricated 3D scaffolds. Furthermore, the excellent solubility of most PPzs in organic solvents allows for casting or electrospinning. For example, the feasibility of electrospinning tubes of poly[(ethyl phenylalanato)_1.4_ (ethyl glycinato)_0.6_ phosphazene] (Figure 44c) has been demonstrated [209] as a model to construct human tissues such as vessels or cardiac valves. Electrospun PLAGA blends can also be prepared for the fabrication of matrices suitable for load-bearing bone regeneration (Figure 44a,b) [210]. Furthermore, coatings have been successfully prepared and investigated; indeed, PCL coated with PPz appears to be one of the most advanced currently under investigation for soft skeletal tissue regeneration [211]. PCL nanofibers coated with PPz ethyl alanine and methylphenol have been shown to improve the ability of the scaffold to support the adhesion and proliferation of stem cells [211,212], showing improvements in the mechanical and morphological properties of the resulting regenerated rotator cuff tendon [211].

Owing to the low glass transition temperatures, which are often below the physiological temperature, PPEs tend to be more suited to coatings, as described above, or soft scaffolds for tissue engineering. For example, the feasibility of PPE-based nerve guide conduits has been demonstrated [214] in a critical-size rat sciatic nerve defect model and shown to be effective aids for nerve regeneration. Further development has brought further improvements in the nerve regeneration through the incorporation of microencapsulated nerve growth factors [215]. Recently, photopolymerized 3D polymer networks have been reported for PPEs proposed as soft implantable medical devices [216]. The authors note the ease of the tunable nature of the system, with important parameters such as the swelling ratio and the degradation rate tailored by the chemical structure and hydrophilicity of the main chains. Furthermore, the modulus could be tailored by the cross-linking density in the soft material range.

Another target therapy for phosphorous-containing polymers is bone tissue engineering. The binding of calcium to phosphate is known to encourage mineral deposition, and indeed polyphosphate shows a high affinity for calcium ions and has been used in bone regeneration [217]. Organic polymers with pendant phosphates have been developed for this purpose [195]. The ability of various polyphosphazenes to mineralize apatite has also been demonstrated, with the assistance of ionically bound calcium, and they have been shown to rapidly form biologically relevant apatite coatings, making them excellent candidates for implantable bone grafts [218]. Furthermore, blends of PPz with poly(α-ester)s have been shown to improve their calcification [206].

**Figure 45:**
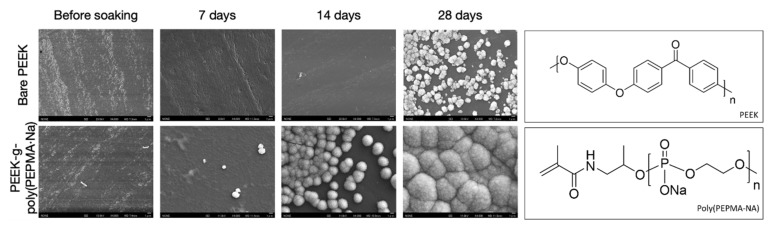
SEM images of pristine polyether ether ketones (PEEK) and PEEK grafted with poly(PEPMA·Na) after soaking in simulated body fluid showing clearly improved deposition for the PPE-coated surfaces. Adapted from [222], with permission from Taylor & Francis.

Meanwhile, PPE–PLA block copolymers have also been shown to increase mineral deposition and the attachment and proliferation of osteoblasts [219]. Indeed, polyphosphoesters have also generated much interest for their strong affinity for biominerals [220], with the ionized phosphodiester unit as the key element exhibiting mineral affinity [220,221]. Polyether ether ketones (PEEK) are clinically used as a replacement for metallic orthopedic implants, but they have been demonstrated to show improved CaP mineral nucleation and biomineralization when coated with PPE (Figure 45) [222]. The sodium salt of an end-functionalized polyphosphoester poly(PEPMA·Na) was grafted to PEEK surfaces, increasing their hydrophilicity, significantly increasing their mineralization, and subsequently improving the proliferation of cells on the modified PEEK surface (Figure 45). This demonstrates the potential of PPE ionomers to significantly improve the osteointegration and hard-tissue compatibility of this important biomedical polymer.

## 5. Conclusions

While main-chain phosphorus-containing polymers have been of interest for therapeutic applications for many decades, there has been a significant expansion of reports in the last 5 years, suggesting a blossoming research field. This is in part due, as described in the first half of this review, to the advent of controlled polymerization routes, which not only allow for controlled molecular weights but also facilitate the preparation of higher architectures and self-assembled nanostructures. A second major driving force of this expansion is the requirement in many therapeutic applications for polymer-based materials that are not only degradable but that can be designed to have suitable degradation rates for the desired applications and the environmental conditions to which they will be exposed. This is underlined in the field of tissue regeneration, whereby the ability to adapt mechanical properties in combination with degradation rates to those of the growing tissue is critical to the success. Likewise, the capability to prepare degradable amphiphilic polymers with finely-tuned LCSTs has invigorated a prospering collection of therapeutics based on degradable injectable hydrogels. Furthermore, it would appear there are some niche applications, where the presence of phosphorus in the polymer main chain has led to unique properties that are not found in organic polymers. For example, the superior adjuvant activity in PPz-based vaccine adjuvants and the antithrombotic effect of PPz-stent coatings, or indeed the superior biomineralization of PPE-coated orthopedic implants. The less acidic degradation products of PPz compared to polyesters are also regarded as a significant feature for degradable implants. In water-soluble applications, the ability to easily functionalize (load with drugs/labels or conjugate to proteins), in direct combination with controlled synthesis, has been widely used to design sophisticated polymer conjugates. The ability to do this whilst at the same time being able to easily tune degradation rates is not found for synthetic organic polymers. All in all, the diverse studies described herein reveal a thriving innovative field researching main-chain phosphorus-containing polymers for therapeutic applications.

## References

[1] Souery W.N., Bishop C.J. (2018). Clinically advancing and promising polymer-based therapeutics. Acta Biomater..

[2] Langer R., Tirrell D.A. (2004). Designing materials for biology and medicine. Nature.

[3] Vidal F., Jäkle F. (2019). Funktionelle polymere Materialien auf der Basis von Hauptgruppen-Elementen. Angew. Chem..

[4] Westheimer F. (1987). Why nature chose phosphates. Science.

[5] Emsley J. (2000). The 13th Element: The Sordid Tale of Murder.

[6] Hey-Hawkins E. Phosphorus—The Devil’s Element?. Proceedings of the 18th Austrian Chemistry Days.

[7] Monge S., Canniccioni B., Graillot A., Robin J.-J. (2011). Phosphorus-Containing Polymers: A Great Opportunity for the Biomedical Field. Biomacromolecules.

[8] Velencoso M.M., Battig A., Markwart J.C., Schartel B., Wurm F.R. (2018). Molecular Firefighting—How Modern Phosphorus Chemistry Can Help Solve the Challenge of Flame Retardancy. Angew. Chem. Int. Ed..

[9] Carriedo G.A., Alonso F.L.G., Gomez-Elipe P., Fidalgo J.I., Alvarez J.L.G., Presa-Soto A. (2003). A simplified and convenient laboratory-scale preparation of N-14 or N-15 high molecular weight poly(dichlorophosphazene) directly from PCl5. Chem. Eur. J..

[10] Andrianov A.K., Chen J., LeGolvan M.P. (2004). Poly(dichlorophosphazene) As a Precursor for Biologically Active Polyphosphazenes: Synthesis, Characterization, and Stabilization. Macromolecules.

[11] Tian Z., Hess A., Fellin C.R., Nulwala H., Allcock H.R. (2015). Phosphazene High Polymers and Models with Cyclic Aliphatic Side Groups: New Structure–Property Relationships. Macromolecules.

[12] Steinbach T., Wurm F.R. (2015). Poly(phosphoester)s: A New Platform for Degradable Polymers. Angew. Chem. Int. Ed..

[13] Rothemund S., Teasdale I. (2016). Preparation of polyphosphazenes: A tutorial review. Chem. Soc. Rev..

[14] Allcock H.R., Crane C.A., Morrissey C.T., Nelson J.M., Reeves S.D., Honeyman C.H., Manners I. (1996). “Living” cationic polymerization of phosphoranimines as an ambient temperature route to polyphosphazenes with controlled molecular weights. Macromolecules.

[15] Honeyman C.H., Manners I., Morrissey C.T., Allcock H.R. (1995). Ambient Temperature Synthesis of Poly(dichlorophosphazene) with Molecular Weight Control. J. Am. Chem. Soc..

[16] Wang B. (2005). Development of a one-pot in situ synthesis of poly(dichlorophosphazene) from PCl3. Macromolecules.

[17] Suárez Suárez S., Presa Soto D., Carriedo G.A., Presa Soto A., Staubitz A. (2012). Experimental and Theoretical Study of the Living Polymerization of N-Silylphosphoranimines. Synthesis of New Block Copolyphosphazenes. Organometallics.

[18] Wilfert S., Henke H., Schoefberger W., Brüggemann O., Teasdale I. (2014). Chain-End-Functionalized Polyphosphazenes via a One-Pot Phosphine-Mediated Living Polymerization. Macromol. Rapid Commun..

[19] Carriedo G.A., de la Campa R., Soto A.P. (2018). Polyphosphazenes – Synthetically Versatile Block Copolymers (“Multi-Tool”) for Self-Assembly. Eur. J. Inorg. Chem..

[20] Soto A.P., Manners I. (2009). Poly(ferrocenylsilane-b-polyphosphazene) (PFS-b-PP): A New Class of Organometallic-Inorganic Block Copolymers. Macromolecules.

[21] Henke H., Brüggemann O., Teasdale I. (2017). Branched Macromolecular Architectures for Degradable, Multifunctional Phosphorus-Based Polymers. Macromol. Rapid Commun..

[22] Wang Y.-C., Yuan Y.-Y., Du J.-Z., Yang X.-Z., Wang J. (2009). Recent Progress in Polyphosphoesters: From Controlled Synthesis to Biomedical Applications. Macromol. Biosci..

[23] Iwasaki Y., Yamaguchi E. (2010). Synthesis of Well-Defined Thermoresponsive Polyphosphoester Macroinitiators Using Organocatalysts. Macromolecules.

[24] Steinke J.H.G., Greenland B.W., Johns S., Parker M.P., Atkinson R.C.J., Cade I.A., Golding P., Trussell S.J. (2014). Robust and Operationally Simple Synthesis of Poly(bis(2,2,2-trifluoroethoxy) phosphazene) with Controlled Molecular Weight, Low PDI, and High Conversion. ACS Macro Lett..

[25] Steinbach T., Alexandrino E.M., Wurm F.R. (2013). Unsaturated poly(phosphoester)s via ring-opening metathesis polymerization. Polym. Chem..

[26] Steinmann M., Markwart J., Wurm F.R. (2014). Poly(alkylidene chlorophosphate)s via Acyclic Diene Metathesis Polymerization: A General Platform for the Postpolymerization Modification of Poly(phosphoester)s. Macromolecules.

[27] Steinmann M., Wagner M., Wurm F.R. (2016). Poly(phosphorodiamidate)s by Olefin Metathesis Polymerization with Precise Degradation. Chem. Eur. J..

[28] Wisian-Neilson P., Neilson R.H. (2018). Synthesis and Modification of Poly(alkyl/arylphosphazenes). Polyphosphazenes in Biomedicine, Engineering, and Pioneering Synthesis.

[29] Steinbach T., Ritz S., Wurm F.R. (2014). Water-Soluble Poly(phosphonate)s via Living Ring-Opening Polymerization. ACS Macro Lett..

[30] Wolf T., Steinbach T., Wurm F.R. (2015). A Library of Well-Defined and Water-Soluble Poly(alkyl phosphonate)s with Adjustable Hydrolysis. Macromolecules.

[31] Yilmaz Z.E., Jérôme C. (2016). Polyphosphoesters: New Trends in Synthesis and Drug Delivery Applications. Macromol. Biosci..

[32] Henke H., Wilfert S., Iturmendi A., Brueggemann O., Teasdale I. (2013). Branched Polyphosphazenes with Controlled Dimensions. J. Polym. Sci. A Polym. Chem..

[33] Zhang S., Li A., Zou J., Lin L.Y., Wooley K.L. (2012). Facile Synthesis of Clickable, Water-Soluble, and Degradable Polyphosphoesters. ACS Macro Lett..

[34] Chen C., Xu H., Qian Y.-C., Huang X.-J. (2015). Glycosylation of polyphosphazenes by thiol-yne click chemistry for lectin recognition. RSC Adv..

[35] Bouché M., Pühringer M., Iturmendi A., Amirshaghaghi A., Tsourkas A., Teasdale I., Cormode D.P. (2019). Activatable Hybrid Polyphosphazene-AuNP Nanoprobe for ROS Detection by Bimodal PA/CT Imaging. ACS Appl. Mater. Interfaces.

[36] Qian Y., Huang X., Xu Z. (2015). Synthesis of Polyphosphazene Derivatives via Thiol-ene Click Reactions in an Aqueous Medium. Macromol. Chem. Phys..

[37] Huang X., Huang X.-J., Yu A.-G., Wang C., Dai Z.-W., Xu Z.-K. (2011). “Click Chemistry” as a Facile Approach to the Synthesis of Polyphosphazene Glycopolymers. Macromol. Chem. Phys..

[38] Moura L.I.F., Malfanti A., Peres C., Matos A.I., Guegain E., Sainz V., Zloh M., Vicent M.J., Florindo H.F. (2019). Functionalized branched polymers: Promising immunomodulatory tools for the treatment of cancer and immune disorders. Mater. Horiz..

[39] Duro-Castano A., Movellan J., Vicent M.J. (2015). Smart branched polymer drug conjugates as nano-sized drug delivery systems. Biomater. Sci..

[40] Liu X., Tian Z., Chen C., Allcock H.R. (2012). Synthesis and Characterization of Brush-Shaped Hybrid Inorganic/Organic Polymers Based on Polyphosphazenes. Macromolecules.

[41] Henke H., Posch S., Brüggemann O., Teasdale I. (2016). Polyphosphazene Based Star-Branched and Dendritic Molecular Brushes. Macromol. Rapid Commun..

[42] Linhardt A., König M., Iturmendi A., Henke H., Brueggemann O., Teasdale I. (2018). Degradable, dendritic polyols on a branched polyphosphazene backbone. Ind. Eng. Chem. Res..

[43] Liu J., Huang W., Zhou Y., Yan D. (2009). Synthesis of Hyperbranched Polyphosphates by Self-Condensing Ring-Opening Polymerization of HEEP without Catalyst. Macromolecules.

[44] Liu J., Huang W., Pang Y., Yan D. (2015). Hyperbranched polyphosphates: Synthesis, functionalization and biomedical applications. Chem. Soc. Rev..

[45] Battig A., Markwart J.C., Wurm F.R., Schartel B. (2019). Hyperbranched phosphorus flame retardants: Multifunctional additives for epoxy resins. Polym. Chem..

[46] Tauber K., Marsico F., Wurm F.R., Schartel B. (2014). Hyperbranched poly(phosphoester)s as flame retardants for technical and high performance polymers. Polym. Chem..

[47] Caminade A.-M. (2017). Phosphorus dendrimers for nanomedicine. Chem. Commun..

[48] Caminade A.-M., Ouali A., Laurent R., Turrin C.-O., Majoral J.-P. (2015). The dendritic effect illustrated with phosphorus dendrimers. Chem. Soc. Rev..

[49] Caminade A.-M., Majoral J.-P. (2016). Bifunctional Phosphorus Dendrimers and Their Properties. Molecules.

[50] Katir N., El Brahmi N., El Kadib A., Mignani S., Caminade A.-M., Bousmina M., Majoral J.P. (2015). Synthesis of Onion-Peel Nanodendritic Structures with Sequential Functional Phosphorus Diversity. Chem. Eur. J..

[51] Zhai X., Huang W., Liu J., Pang Y., Zhu X., Zhou Y., Yan D. (2011). Micelles from Amphiphilic Block Copolyphosphates for Drug Delivery. Macromol. Biosci..

[52] Aichhorn S., Linhardt A., Halfmann A., Nadlinger M., Kirchberger S., Stadler M., Dillinger B., Distel M., Dohnal A., Teasdale I. (2017). A pH-sensitive Macromolecular Prodrug as TLR7/8 Targeting Immune Response Modifier. Chem. – A Eur. J..

[53] Zhang F., Zhang S., Pollack S.F., Li R., Gonzalez A.M., Fan J., Zou J., Leininger S.E., Pavía-Sanders A., Johnson R. (2015). Improving Paclitaxel Delivery: In Vitro and In Vivo Characterization of PEGylated Polyphosphoester-Based Nanocarriers. J. Am. Chem. Soc..

[54] Qiu L., Fu J. (2018). Applications of Self-Assembled Polyphosphazene Nano-Aggregates in Drug Delivery. Polyphosphazenes in Biomedicine, Engineering, and Pioneering Synthesis.

[55] Linhardt A., König M., Schöfberger W., Brüggemann O., Andrianov A., Teasdale I. (2016). Biodegradable Polyphosphazene Based Peptide-Polymer Hybrids. Polymers.

[56] Zheng C., Qiu L.Y., Zhu K.J. (2009). Novel polymersomes based on amphiphilic graft polyphosphazenes and their encapsulation of water-soluble anti-cancer drug. Polymer.

[57] Suárez-Suárez S., Carriedo G.A., Presa Soto A. (2016). Reversible Morphological Evolution of Responsive Giant Vesicles to Nanospheres by the Self-Assembly of Crystalline-b-Coil Polyphosphazene Block Copolymers. Chem. – A Eur. J..

[58] Wang F., Wang Y.-C., Yan L.-F., Wang J. (2009). Biodegradable vesicular nanocarriers based on poly(ɛ-caprolactone)-block-poly(ethyl ethylene phosphate) for drug delivery. Polymer.

[59] Rideau E., Wurm F.R., Landfester K. (2018). Giant polymersomes from non-assisted film hydration of phosphate-based block copolymers. Polym. Chem..

[60] Wilfert S., Iturmendi A., Henke H., Brüggemann O., Teasdale I. (2014). Thermoresponsive Polyphosphazene-Based Molecular Brushes by Living Cationic Polymerization. Macromol. Symp..

[61] Kneidinger M., Iturmendi A., Ulbricht C., Truglas T., Groiss H., Teasdale I., Salinas Y. (2019). Mesoporous Silica Micromotors with a Reversible Temperature Regulated On–Off Polyphosphazene Switch. Macromol. Rapid Commun..

[62] Hong K.H., Kim Y.-M., Song S.-C. (2019). Fine-Tunable and Injectable 3D Hydrogel for On-Demand Stem Cell Niche. Adv. Sci..

[63] Seo B.-B., Koh J.-T., Song S.-C. (2017). Tuning physical properties and BMP-2 release rates of injectable hydrogel systems for an optimal bone regeneration effect. Biomaterials.

[64] Kim Y.-M., Potta T., Park K.-H., Song S.-C. (2017). Temperature responsive chemical crosslinkable UV pretreated hydrogel for application to injectable tissue regeneration system via differentiations of encapsulated hMSCs. Biomaterials.

[65] Zhang Z.-Q., Song S.-C. (2017). Multiple hyperthermia-mediated release of TRAIL/SPION nanocomplex from thermosensitive polymeric hydrogels for combination cancer therapy. Biomaterials.

[66] Seo B.-B., Choi H., Koh J.-T., Song S.-C. (2015). Sustained BMP-2 delivery and injectable bone regeneration using thermosensitive polymeric nanoparticle hydrogel bearing dual interactions with BMP-2. J. Control. Release.

[67] Cho J.-K., Hong J.M., Han T., Yang H.-K., Song S.-C. (2013). Injectable and biodegradable poly(organophosphazene) hydrogel as a delivery system of docetaxel for cancer treatment. J. Drug Target..

[68] Park M.-R., Seo B.-B., Song S.-C. (2013). Dual ionic interaction system based on polyelectrolyte complex and ionic, injectable, and thermosensitive hydrogel for sustained release of human growth hormone. Biomaterials.

[69] Seo B.-B., Park M.-R., Chun C., Lee J.-Y., Song S.-C. (2011). The biological efficiency and bioavailability of human growth hormone delivered using injectable, ionic, thermosensitive poly(organophosphazene)-polyethylenimine conjugate hydrogels. Biomaterials.

[70] Potta T., Chun C., Song S.-C. (2010). Injectable, dual cross-linkable polyphosphazene blend hydrogels. Biomaterials.

[71] Potta T., Chun C., Song S.C. (2009). Chemically crosslinkable thermosensitive polyphosphazene gels as injectable materials for biomedical applications. Biomaterials.

[72] Kim Y.-M., Park M.-R., Song S.-C. (2012). Injectable Polyplex Hydrogel for Localized and Long-Term Delivery of siRNA. ACS Nano.

[73] Iwasaki Y., Wachiralarpphaithoon C., Akiyoshi K. (2007). Novel Thermoresponsive Polymers Having Biodegradable Phosphoester Backbones. Macromolecules.

[74] Wolf T., Hunold J., Simon J., Rosenauer C., Hinderberger D., Wurm F.R. (2018). Temperature responsive poly(phosphonate) copolymers: From single chains to macroscopic coacervates. Polym. Chem..

[75] Becker G., Marquetant T.A., Wagner M., Wurm F.R. (2017). Multifunctional Poly(phosphoester)s for Reversible Diels–Alder Postmodification To Tune the LCST in Water. Macromolecules.

[76] Wolf T., Rheinberger T., Simon J., Wurm F.R. (2017). Reversible Self-Assembly of Degradable Polymersomes with Upper Critical Solution Temperature in Water. J. Am. Chem. Soc..

[77] Gustafson T.P., Lonnecker A.T., Heo G.S., Zhang S., Dove A.P., Wooley K.L. (2013). Poly(d-glucose carbonate) Block Copolymers: A Platform for Natural Product-Based Nanomaterials with Solvothermatic Characteristics. Biomacromolecules.

[78] Markovsky E., Baabur-Cohen H., Eldar-Boock A., Omer L., Tiram G., Ferber S., Ofek P., Polyak D., Scomparin A., Satchi-Fainaro R. (2012). Administration, distribution, metabolism and elimination of polymer therapeutics. J. Control. Release.

[79] Allcock H.R., Morozowich N.L. (2012). Bioerodible polyphosphazenes and their medical potential. Polym. Chem..

[80] Wilfert S., Iturmendi A., Schoefberger W., Kryeziu K., Heffeter P., Berger W., Brüggemann O., Teasdale I. (2014). Water-soluble, biocompatible polyphosphazenes with controllable and pH-promoted degradation behavior. J. Polym. Sci. A Polym. Chem..

[81] DeCollibus D.P., Marin A., Andrianov A.K. (2010). Effect of Environmental Factors on Hydrolytic Degradation of Water-Soluble Polyphosphazene Polyelectrolyte in Aqueous Solutions. Biomacromolecules.

[82] Bauer K.N., Liu L., Wagner M., Andrienko D., Wurm F.R. (2018). Mechanistic study on the hydrolytic degradation of polyphosphates. Eur. Polym. J..

[83] Wang H., Su L., Li R., Zhang S., Fan J., Zhang F., Nguyen T.P., Wooley K.L. (2017). Polyphosphoramidates That Undergo Acid-Triggered Backbone Degradation. ACS Macro Lett..

[84] Bates M.C., Yousaf A., Sun L., Barakat M., Kueller A. (2019). Translational Research and Early Favorable Clinical Results of a Novel Polyphosphazene (Polyzene-F) Nanocoating. Regen. Eng. Transl. Med..

[85] Hiroyuki J., Hiroyoshi M., Qi C., Matthew K., Sho T., Atsushi S., Liang G., Eduardo A., Anuj G., Frank D.K. (2019). Thromboresistance and functional healing in the COBRA PzF stent versus competitor DES: Implications for dual antiplatelet therapy. EuroIntervention.

[86] Sakakura K., Cheng Q., Otsuka F., Yahagi K., Barakat M., Ren J., Ladich E., Kolodgie F.D., Virmani R. (2013). TCT-806 Thrombogenicity Of Novel Polyphosphazene Surface-modified Coronary Stent Compared To Standard Bare Metal Stent In Swine Shunt Model. J. Am. Coll. Cardiol..

[87] Henn C., Satzl S., Christoph P., Kurz P., Radeleff B., Stampfl U., Stampfl S., Berger I., Richter G.M. (2008). Efficacy of a Polyphosphazene Nanocoat in Reducing Thrombogenicity, In-stent Stenosis, and Inflammatory Response in Porcine Renal and Iliac Artery Stents. J. Vasc. Interv. Radiol..

[88] Andrianov A.K. (2018). Self-Assembling Ionic Polyphosphazenes and Their Biomedical Applications. Polyphosphazenes in Biomedicine, Engineering, and Pioneering Synthesis.

[89] Andrianov A.K. (2009). Polyphosphazenes for Biomedical Applications.

[90] Ogueri K.S., Ivirico J.L.E., Nair L.S., Allcock H.R., Laurencin C.T. (2017). Biodegradable Polyphosphazene-Based Blends for Regenerative Engineering. Regen. Eng. Transl. Med..

[91] Su L., Li R., Khan S., Clanton R., Zhang F., Lin Y.-N., Song Y., Wang H., Fan J., Hernandez S. (2018). Chemical Design of Both a Glutathione-Sensitive Dimeric Drug Guest and a Glucose-Derived Nanocarrier Host to Achieve Enhanced Osteosarcoma Lung Metastatic Anticancer Selectivity. J. Am. Chem. Soc..

[92] Liu S., Maheshwari R., Kiick K.L. (2009). Polymer-Based Therapeutics. Macromolecules.

[93] Duncan R. (2014). Polymer therapeutics: Top 10 selling pharmaceuticals—What next?. J. Control. Release.

[94] Kopeček J. (2013). Polymer–drug conjugates: Origins, progress to date and future directions. Adv. Drug Deliv. Rev..

[95] Yang J., Kopeček J. (2017). The Light at the End of the Tunnel-Second Generation HPMA Conjugates for Cancer Treatment. Curr. Opin. Colloid Interface Sci..

[96] Duncan R. (2006). Polymer conjugates as anticancer nanomedicines. Nat. Rev. Cancer.

[97] Hackl C.M., Schoenhacker-Alte B., Klose M.H.M., Henke H., Legina M.S., Jakupec M.A., Berger W., Keppler B.K., Bruggemann O., Teasdale I. (2017). Synthesis and in vivo anticancer evaluation of poly(organo)phosphazene-based metallodrug conjugates. Dalton Trans..

[98] Henke H., Kryeziu K., Banfić J., Theiner S., Körner W., Brüggemann O., Berger W., Keppler B.K., Heffeter P., Teasdale I. (2016). Macromolecular Pt(IV) Prodrugs from Poly(organo)phosphazenes. Macromol. Biosci..

[99] Avaji P.G., Joo H.I., Park J.H., Park K.S., Jun Y.J., Lee H.J., Sohn Y.S. (2014). Synthesis and properties of a new micellar polyphosphazene–platinum(II) conjugate drug. J. Inorg. Biochem..

[100] Jun Y.J., Kim J.I., Jun M.J., Sohn Y.S. (2005). Selective tumor targeting by enhanced permeability and retention effect. Synthesis and antitumor activity of polyphosphazene-platinum (II) conjugates. J. Inorg. Biochem..

[101] Song R., Jun Y.J., Kim J.I., Jin C., Sohn Y.S. (2005). Synthesis, characterization, and tumor selectivity of a polyphosphazene-platinum(II) conjugate. J. Control. Release.

[102] Avaji P.G., Park J.H., Lee H.J., Jun Y.J., Park K.S., Lee K.E., Choi S.J., Lee H.J., Sohn Y.S. (2016). Design of a novel theranostic nanomedicine: Synthesis and physicochemical properties of a biocompatible polyphosphazene-platinum(II) conjugate. Int. J. Nanomed..

[103] Mitova V., Slavcheva S., Shestakova P., Momekova D., Stoyanov N., Momekov G., Troev K., Koseva N. (2014). Polyphosphoester conjugates of dinuclear platinum complex: Synthesis and evaluation of cytotoxic and the proapoptotic activity. Eur. J. Med. Chem..

[104] Sun C.-Y., Dou S., Du J.-Z., Yang X.-Z., Li Y.-P., Wang J. (2014). Doxorubicin Conjugate of Poly(Ethylene Glycol)- Block -Polyphosphoester for Cancer Therapy. Adv. Healthc. Mater..

[105] Liu J., Huang W., Pang Y., Zhu X., Zhou Y., Yan D. (2010). Hyperbranched Polyphosphates for Drug Delivery Application: Design, Synthesis, and In Vitro Evaluation. Biomacromolecules.

[106] Shcharbin D., Dzmitruk V., Shakhbazau A., Goncharova N., Seviaryn I., Kosmacheva S., Potapnev M., Pedziwiatr-Werbicka E., Bryszewska M., Talabaev M. (2011). Fourth Generation Phosphorus-Containing Dendrimers: Prospective Drug and Gene Delivery Carrier. Pharmaceutics.

[107] Blanzat M., Turrin C.-O., Aubertin A.-M., Couturier-Vidal C., Caminade A.-M., Majoral J.-P., Rico-Lattes I., Lattes A. (2005). Dendritic Catanionic Assemblies: In vitro Anti-HIV Activity of Phosphorus-Containing Dendrimers Bearing Galβ1cer Analogues. ChemBioChem.

[108] El Brahmi N., El Kazzouli S., Mignani S.M., Essassi E.M., Aubert G., Laurent R., Caminade A.-M., Bousmina M.M., Cresteil T., Majoral J.-P. (2013). Original Multivalent Copper(II)-Conjugated Phosphorus Dendrimers and Corresponding Mononuclear Copper(II) Complexes with Antitumoral Activities. Mol. Pharm..

[109] Ciepluch K., Katir N., El Kadib A., Felczak A., Zawadzka K., Weber M., Klajnert B., Lisowska K., Caminade A.-M., Bousmina M. (2012). Biological Properties of New Viologen-Phosphorus Dendrimers. Mol. Pharm..

[110] Servin P., Laurent R., Tristany M., Romerosa A., Peruzzini M., Garcia-Maroto F., Majoral J.P., Caminade A.M. (2018). Dual properties of water-soluble Ru-PTA complexes of dendrimers: Catalysis and interaction with DNA. Inorg. Chim. Acta.

[111] El Brahmi N., Mignani S.M., Caron J., El Kazzouli S., Bousmina M.M., Caminade A.M., Cresteil T., Majoral J.P. (2015). Investigations on dendrimer space reveal solid and liquid tumor growth-inhibition by original phosphorus-based dendrimers and the corresponding monomers and dendrons with ethacrynic acid motifs. Nanoscale.

[112] Mignani S., El Brahmi N., Eloy L., Poupon J., Nicolas V., Steinmetz A., El Kazzouli S., Bousmina M.M., Blanchard-Desce M., Caminade A.M. (2017). Anticancer copper(II) phosphorus dendrimers are potent proapoptotic Bax activators. Eur. J. Med. Chem..

[113] Caminade A.-M. (2016). Inorganic dendrimers: Recent advances for catalysis, nanomaterials, and nanomedicine. Chem. Soc. Rev..

[114] Amin M.C.I.M., Butt A.M., Amjad M.W., Kesharwani P. (2017). Polymeric Micelles for Drug Targeting and Delivery. Nanotechnology-Based Approaches for Targeting and Delivery of Drugs and Genes.

[115] Meerovich I., Dash A.K. (2019). Polymersomes for drug delivery and other biomedical applications. Materials for Biomedical Engineering.

[116] Peng Y., Zhu X., Qiu L. (2016). Electroneutral composite polymersomes self-assembled by amphiphilic polyphosphazenes for effective miR-200c in vivo delivery to inhibit drug resistant lung cancer. Biomaterials.

[117] Fu J., Liang L., Qiu L. (2017). In Situ Generated Gold Nanoparticle Hybrid Polymersomes for Water-Soluble Chemotherapeutics: Inhibited Leakage and pH-Responsive Intracellular Release. Adv. Funct. Mater..

[118] Mehnath S., Arjama M., Rajan M., Jeyaraj M. (2018). Development of cholate conjugated hybrid polymeric micelles for FXR receptor mediated effective site-specific delivery of paclitaxel. New J. Chem..

[119] Marsico F., Wagner M., Landfester K., Wurm F.R. (2012). Unsaturated polyphosphoesters via acyclic diene metathesis polymerization. Macromolecules.

[120] Alexandrino E.M., Ritz S., Marsico F., Baier G., Mailänder V., Landfester K., Wurm F.R. (2014). Paclitaxel-loaded polyphosphate nanoparticles: A potential strategy for bone cancer treatment. J. Mater. Chem. B.

[121] Yilmaz Z.E., Vanslambrouck S., Cajot S., Thiry J., Debuigne A., Lecomte P., Jérôme C., Riva R. (2016). Core cross-linked micelles of polyphosphoester containing amphiphilic block copolymers as drug nanocarriers. RSC Adv..

[122] Zhang L., Shi D., Shi C., Kaneko T., Chen M. (2019). Supramolecular micellar drug delivery system based on multi-arm block copolymer for highly effective encapsulation and sustained-release chemotherapy. J. Mater. Chem. B.

[123] Yu S., He C., Chen X. (2018). Injectable Hydrogels as Unique Platforms for Local Chemotherapeutics-Based Combination Antitumor Therapy. Macromol. Biosci..

[124] Cirillo G., Spizzirri U.G., Curcio M., Nicoletta F.P., Iemma F. (2019). Injectable Hydrogels for Cancer Therapy over the Last Decade. Pharmaceutics.

[125] Mathew A.P., Uthaman S., Cho K.-H., Cho C.-S., Park I.-K. (2018). Injectable hydrogels for delivering biotherapeutic molecules. Int. J. Biol. Macromol..

[126] Norouzi M., Nazari B., Miller D.W. (2016). Injectable hydrogel-based drug delivery systems for local cancer therapy. Drug Discov. Today.

[127] Cho J.-K., Kuh H.-J., Song S.-C. (2014). Injectable poly(organophosphazene) hydrogel system for effective paclitaxel and doxorubicin combination therapy. J. Drug Target..

[128] Zhang Z.-Q., Kim Y.-M., Song S.-C. (2019). Injectable and Quadruple-Functional Hydrogel as an Alternative to Intravenous Delivery for Enhanced Tumor Targeting. ACS Appl. Mater. Interfaces.

[129] Wang J., Sun D.D.N., Shin-ya Y., Leong K.W. (2004). Stimuli-Responsive Hydrogel Based on Poly(propylene phosphate). Macromolecules.

[130] Li F., He J., Zhang M., Ni P. (2015). A pH-sensitive and biodegradable supramolecular hydrogel constructed from a PEGylated polyphosphoester-doxorubicin prodrug and α-cyclodextrin. Polym. Chem..

[131] Dera R., Diliën H., Billen B., Gagliardi M., Rahimi N., Den Akker N.M.S., Molin D.G.M., Grandfils C., Adriaensens P., Guedens W. (2019). Phosphodiester Hydrogels for Cell Scaffolding and Drug Release Applications. Macromol. Biosci..

[132] Bugaj A.M. (2011). Targeted photodynamic therapy—A promising strategy of tumor treatment. Photochem. Photobiol. Sci..

[133] Xiao P., Zhang J., Zhao J., Stenzel M.H. (2017). Light-induced release of molecules from polymers. Prog. Polym. Sci..

[134] Teasdale I., Waser M., Wilfert S., Falk H., Brüggemann O. (2012). Photoreactive, water-soluble conjugates of hypericin with polyphosphazenes. Monatsh. Chem..

[135] Feinweber D., Verwanger T., Brueggemann O., Teasdale I., Krammer B. (2014). Applicability of new degradable hypericin-polymer-conjugates as photosensitizers: Principal mode of action demonstrated by in vitro models. Photochem. Photobiol. Sci..

[136] Iturmendi A., Theis S., Maderegger D., Monkowius U., Teasdale I. (2018). Coumarin-Caged Polyphosphazenes with a Visible-Light Driven On-Demand Degradation. Macromol. Rapid Commun..

[137] Li F., Chen C., Yang X., He X., Zhao Z., Li J., Yu Y., Yang X., Wang J. (2018). Acetal-Linked Hyperbranched Polyphosphoester Nanocarriers Loaded with Chlorin e6 for pH-Activatable Photodynamic Therapy. ACS Appl. Mater. Interfaces.

[138] Pei P., Sun C., Tao W., Li J., Yang X., Wang J. (2019). ROS-sensitive thioketal-linked polyphosphoester-doxorubicin conjugate for precise phototriggered locoregional chemotherapy. Biomaterials.

[139] Moncalvo F., Martinez Espinoza M.I., Cellesi F. (2020). Nanosized Delivery Systems for Therapeutic Proteins: Clinically Validated Technologies and Advanced Development Strategies. Front. Bioeng. Biotechnol..

[140] Barz M., Luxenhofer R., Zentel R., Vicent M.J. (2011). Overcoming the PEG-addiction: Well-defined alternatives to PEG, from structure–property relationships to better defined therapeutics. Polym. Chem..

[141] Pelegri-Oday E.M., Lin E.W., Maynard H.D. (2014). Therapeutic protein-polymer conjugates: Advancing beyond pegylation. J. Am. Chem. Soc..

[142] Andrianov A.K., Marin A., Martinez A.P., Weidman J.L., Fuerst T.R. (2018). Hydrolytically Degradable PEGylated Polyelectrolyte Nanocomplexes for Protein Delivery. Biomacromolecules.

[143] Steinbach T., Wurm F.R. (2016). Degradable Polyphosphoester-Protein Conjugates: "PPEylation" of Proteins. Biomacromolecules.

[144] Pelosi C., Duce C., Russo D., Tiné M.R., Wurm F.R. (2018). PPEylation of proteins: Synthesis, activity, and stability of myoglobin-polyphosphoester conjugates. Eur. Polym. J..

[145] Steinbach T., Becker G., Spiegel A., Figueiredo T., Russo D., Wurm F.R. (2017). Reversible Bioconjugation: Biodegradable Poly(phosphate)-Protein Conjugates. Macromol. Biosci..

[146] Russo D., Plazanet M., Teixeira J., Moulin M., Härtlein M., Wurm F.R., Steinbach T. (2016). Investigation into the Relaxation Dynamics of Polymer-Protein Conjugates Reveals Surprising Role of Polymer Solvation on Inherent Protein Flexibility. Biomacromolecules.

[147] Russo D., De Angelis A., Garvey C.J., Wurm F.R., Appavou M.S., Prevost S. (2019). Effect of Polymer Chain Density on Protein-Polymer Conjugate Conformation. Biomacromolecules.

[148] Russo D., De Angelis A., Paciaroni A., Frick B., De Sousa N., Wurm F.R., Teixeira J. (2019). Protein-Polymer Dynamics as Affected by Polymer Coating and Interactions. Langmuir.

[149] Duncan R., Richardson S.C.W. (2012). Endocytosis and Intracellular Trafficking as Gateways for Nanomedicine Delivery: Opportunities and Challenges. Mol. Pharm..

[150] Martinez A.P., Qamar B., Fuerst T.R., Muro S., Andrianov A.K. (2017). Biodegradable “Smart” Polyphosphazenes with Intrinsic Multifunctionality as Intracellular Protein Delivery Vehicles. Biomacromolecules.

[151] Andrianov A.K., Marin A., Fuerst T.R. (2016). Self-assembly of polyphosphazene immunoadjuvant with poly(ethylene oxide) enables advanced nanoscale delivery modalities and regulated pH-dependent cellular membrane activity. Heliyon.

[152] Hsu W.-H., Sánchez-Gómez P., Gomez-Ibarlucea E., Ivanov D.P., Rahman R., Grabowska A.M., Csaba N., Alexander C., Garcia-Fuentes M. (2019). Structure-Optimized Interpolymer Polyphosphazene Complexes for Effective Gene Delivery against Glioblastoma. Adv. Ther..

[153] Luten J., van Nostruin C.F., De Smedt S.C., Hennink W.E. (2008). Biodegradable polymers as non-viral carriers for plasmid DNA delivery. J. Control. Release.

[154] de Wolf H.K., de Raad M., Snel C., van Steenbergen M.J., Fens M., Storm G., Hennink W.E. (2007). Biodegradable poly(2-dimethylamino ethylamino)phosphazene for in vivo gene delivery to tumor cells. Effect of polymer molecular weight. Pharm. Res..

[155] Hsu W.-H., Csaba N., Alexander C., Garcia-Fuentes M. (2019). Polyphosphazenes for the delivery of biopharmaceuticals. J. Appl. Polymer Sci..

[156] Kaufman H.L., Atkins M.B., Subedi P., Wu J., Chambers J., Joseph Mattingly T., Campbell J.D., Allen J., Ferris A.E., Schilsky R.L. (2019). The promise of Immuno-oncology: Implications for defining the value of cancer treatment. J. Immunother. Cancer.

[157] Xiang Y., Oo N.N.L., Lee J.P., Li Z., Loh X.J. (2017). Recent development of synthetic nonviral systems for sustained gene delivery. Drug Discov. Today.

[158] Gao M., Zhu X., Wu L., Qiu L. (2016). Cationic Polyphosphazene Vesicles for Cancer Immunotherapy by Efficient in Vivo Cytokine IL-12 Plasmid Delivery. Biomacromolecules.

[159] Fan J., He Q., Jin Z., Chen W., Huang W. (2018). A novel phosphoester-based cationic co-polymer nanocarrier delivers chimeric antigen receptor plasmid and exhibits anti-tumor effect. RSC Advances.

[160] Paulis L.E., Mandal S., Kreutz M., Figdor C.G. (2013). Dendritic cell-based nanovaccines for cancer immunotherapy. Curr. Opin. Immunol..

[161] Schöttler S., Becker G., Winzen S., Steinbach T., Mohr K., Landfester K., Mailänder V., Wurm F.R. (2016). Protein adsorption is required for stealth effect of poly(ethylene glycol)- and poly(phosphoester)-coated nanocarriers. Nat. Nano.

[162] Müller J., Bauer K.N., Prozeller D., Simon J., Mailänder V., Wurm F.R., Winzen S., Landfester K. (2017). Coating nanoparticles with tunable surfactants facilitates control over the protein corona. Biomaterials.

[163] Simon J., Wolf T., Klein K., Landfester K., Wurm F.R., Mailänder V. (2018). Hydrophilicity Regulates the Stealth Properties of Polyphosphoester-Coated Nanocarriers. Angew. Chem. Int. Ed..

[164] Simon J., Bauer K.N., Langhanki J., Opatz T., Mailänder V., Landfester K., Wurm F.R. (2019). Noncovalent Targeting of Nanocarriers to Immune Cells with Polyphosphoester-Based Surfactants in Human Blood Plasma. Adv. Sci..

[165] Poupot M., Turrin C.O., Caminade A.M., Fournié J.J., Attal M., Poupot R., Fruchon S. (2016). Poly(phosphorhydrazone) dendrimers: Yin and yang of monocyte activation for human NK cell amplification applied to immunotherapy against multiple myeloma. Nanomed. Nanotechnol. Biol. Med..

[166] Childs R.W., Berg M. (2013). Bringing natural killer cells to the clinic: Ex vivo manipulation. Hematology. Am. Soc. Hematology. Educ. Program.

[167] Andrianov A.K., Marin A., Chen J. (2005). Synthesis, Properties, and Biological Activity of Poly[di(sodium carboxylatoethylphenoxy)phosphazene]. Biomacromolecules.

[168] Andrianov A.K. (2006). Water-soluble polyphosphazenes for biomedical applications. J. Inorg. Organomet. Polym. Mater..

[169] Andrianov A.K. (2006). Polyphosphazenes as Vaccine Adjuvants. Vaccine Adjuvants and Delivery Systems.

[170] Andrianov A.K., Sargent J.R., Sule S.S., Le Golvan M.P., Woods A.L., Jenkins S.A., Payne L.G. (1998). Synthesis, Physico-Chemical Properties and Immunoadjuvant Activity of Water-Soluble Phosphazene Polyacids. J. Bioact. Compat. Polym..

[171] Payne L., Jenkins S., Andrianov A., Roberts B., Powell M., Newman M. (1995). Water-Soluble Phosphazene Polymers for Parenteral and Mucosal Vaccine Delivery. Vaccine Design.

[172] Andrianov A.K., Marin A., Fuerst T.R. (2016). Molecular-Level Interactions of Polyphosphazene Immunoadjuvants and Their Potential Role in Antigen Presentation and Cell Stimulation. Biomacromolecules.

[173] Shakya A.K., Nandakumar K.S. (2013). Applications of polymeric adjuvants in studying autoimmune responses and vaccination against infectious diseases. J. R. Soc. Interface.

[174] Magiri R., Mutwiri G., Wilson H.L. (2018). Recent advances in experimental polyphosphazene adjuvants and their mechanisms of action. Cell Tissue Res..

[175] Dar A., Lai K., Dent D., Potter A., Gerdts V., Babiuk L.A., Mutwiri G.K. (2012). Administration of poly di(sodium carboxylatoethylphenoxy) phosphazene (PCEP) as adjuvant activated mixed Th1/Th2 immune responses in pigs. Vet. Immunol. Immunopathol..

[176] Lu Y., Salvato M.S., Pauza C.D., Li J., Sodroski J., Manson K., Wyand M., Letvin N., Jenkins S., Touzjian N. (1996). Utility of SHIV for Testing HIV-1 Vaccine Candidates in Macaques. J. Acquir. Immune Defic. Syndr..

[177] Le Cam N.N.B., Ronco J., Francon A., Blondeau C., Fanget B. (1998). Adjuvants for influenza vaccine. Res. Immunol..

[178] Payne L.G., Van Nest G., Barchfeld G.L., Siber G.R., Gupta R.K., Jenkins S.A. (1998). PCPP as a parenteral adjuvant for diverse antigens. Dev. Biol. Stand..

[179] Andrianov A.K., DeCollibus D.P., Gillis H.A., Kha H.H., Marin A., Prausnitz M.R., Babiuk L.A., Townsend H., Mutwiri G. (2009). Poly[di(carboxylatophenoxy)phosphazene] is a potent adjuvant for intradermal immunization. Proc. Natl. Acad. Sci. USA.

[180] Andrianov A.K., Mutwiri G. (2012). Intradermal immunization using coated microneedles containing an immunoadjuvant. Vaccine.

[181] Ling L.L., Schneider T., Peoples A.J., Spoering A.L., Engels I., Conlon B.P., Mueller A., Schäberle T.F., Hughes D.E., Epstein S. (2015). A new antibiotic kills pathogens without detectable resistance. Nature.

[182] Lim Y.H., Tiemann K.M., Heo G.S., Wagers P.O., Rezenom Y.H., Zhang S., Zhang F., Youngs W.J., Hunstad D.A., Wooley K.L. (2015). Preparation and in Vitro Antimicrobial Activity of Silver-Bearing Degradable Polymeric Nanoparticles of Polyphosphoester-block-Poly(l-lactide). ACS Nano.

[183] Shah P.N., Shah K.N., Smolen J.A., Tagaev J.A., Torrealba J., Zhou L., Zhang S., Zhang F., Wagers P.O., Panzner M.J. (2018). A novel in vitro metric predicts in vivo efficacy of inhaled silver-based antimicrobials in a murine Pseudomonas aeruginosa pneumonia model. Sci. Rep..

[184] Aweda T.A., Zhang S., Mupanomunda C., Burkemper J., Heo G.S., Bandara N., Lin M., Cutler C.S., Cannon C.L., Youngs W.J. (2015). Investigating the pharmacokinetics and biological distribution of silver-loaded polyphosphoester-based nanoparticles using 111Ag as a radiotracer. J. Label. Compd. Radiopharm..

[185] Pranantyo D., Xu L.Q., Kang E.-T., Mya M.K., Chan-Park M.B. (2016). Conjugation of Polyphosphoester and Antimicrobial Peptide for Enhanced Bactericidal Activity and Biocompatibility. Biomacromolecules.

[186] Cheheltani R., Ezzibdeh R.M., Chhour P., Pulaparthi K., Kim J., Jurcova M., Hsu J.C., Blundell C., Litt H.I., Ferrari V.A. (2016). Tunable, biodegradable gold nanoparticles as contrast agents for computed tomography and photoacoustic imaging. Biomaterials.

[187] Kim J., Silva A.B., Hsu J.C., Maidment P.S.N., Shapira N., Noël P.B., Cormode D.P. (2020). Radioprotective Garment-Inspired Biodegradable Polymetal Nanoparticles for Enhanced CT Contrast Production. Chem. Mater..

[188] Razavi R., Khan Z., Haeberle C.B., Beam D. (1993). Clinical Applications of a Polyphosphazene-Based Resilient Denture Liner. J. Prosthodont..

[189] Xu L.-C., Li Z., Tian Z., Chen C., Allcock H.R., Siedlecki C.A. (2018). A new textured polyphosphazene biomaterial with improved blood coagulation and microbial infection responses. Acta Biomater..

[190] Selin V., Albright V., Ankner J.F., Marin A., Andrianov A.K., Sukhishvili S.A. (2018). Biocompatible Nanocoatings of Fluorinated Polyphosphazenes through Aqueous Assembly. ACS Appl. Mater. Interfaces.

[191] Albright V., Marin A., Kaner P., Sukhishvili S.A., Andrianov A.K. (2019). New Family of Water-Soluble Sulfo–Fluoro Polyphosphazenes and Their Assembly within Hemocompatible Nanocoatings. ACS Appl. Bio Mater..

[192] Lutzke A., Neufeld B.H., Neufeld M.J., Reynolds M.M. (2016). Nitric oxide release from a biodegradable cysteine-based polyphosphazene. J. Mater. Chem. B.

[193] Lutzke A., Tapia J.B., Neufeld M.J., Reynolds M.M. (2017). Sustained Nitric Oxide Release from a Tertiary S-Nitrosothiol-based Polyphosphazene Coating. ACS Appl. Mater. Interfaces.

[194] Place E.S., George J.H., Williams C.K., Stevens M.M. (2009). Synthetic polymer scaffolds for tissue engineering. Chem. Soc. Rev..

[195] Watson B.M., Kasper F.K., Mikos A.G. (2014). Phosphorous-containing polymers for regenerative medicine. Biomed. Mater..

[196] Laurencin C.T., Norman M.E., Elgendy H.M., El-Amin S.F., Allcock H.R., Pucher S.R., Ambrosio A.A. (1993). Use of polyphosphazenes for skeletal tissue regeneration. J. Biomed. Mater. Res..

[197] Deng M., Kumbar S.G., Wan Y., Toti U.S., Allcock H.R., Laurencin C.T. (2010). Polyphosphazene polymers for tissue engineering: An analysis of material synthesis, characterization and applications. Soft Matter.

[198] Peach M.S., Kumbar S.G., James R., Toti U.S., Balasubramaniam D., Deng M., Ulery B., Mazzocca A.D., McCarthy M.B., Morozowich N.L. (2012). Design and Optimization of Polyphosphazene Functionalized Fiber Matrices for Soft Tissue Regeneration. J. Biomed. Nanotechnol..

[199] Saveh-Shemshaki N., Nair L.S., Laurencin C.T. (2019). Nanofiber-based matrices for rotator cuff regenerative engineering. Acta Biomater..

[200] Huang Z., Yang L., Hu X., Huang Y., Cai Q., Ao Y., Yang X. (2019). Molecular Mechanism Study on Effect of Biodegradable Amino Acid Ester–Substituted Polyphosphazenes in Stimulating Osteogenic Differentiation. Macromol. Biosci..

[201] Ambrosio A.M.A., Allcock H.R., Katti D.S., Laurencin C.T. (2002). Degradable polyphosphazene/poly(α-hydroxyester) blends: Degradation studies. Biomaterials.

[202] Sethuraman S., Nair L.S., El-Amin S., Nguyen M.T., Singh A., Krogman N., Greish Y.E., Allcock H.R., Brown P.W., Laurencin C.T. (2010). Mechanical properties and osteocompatibility of novel biodegradable alanine based polyphosphazenes: Side group effects. Acta Biomater..

[203] Singh A., Krogman N.R., Sethuraman S., Nair L.S., Sturgeon J.L., Brown P.W., Laurencin C.T., Allcock H.R. (2006). Effect of Side Group Chemistry on the Properties of Biodegradable l-Alanine Cosubstituted Polyphosphazenes. Biomacromolecules.

[204] Ogueri K.S., Allcock H.R., Laurencin C.T. (2019). Generational biodegradable and regenerative polyphosphazene polymers and their blends with poly (lactic-co-glycolic acid). Prog. Polym. Sci..

[205] Weikel A.L., Cho S.Y., Morozowich N.L., Nair L.S., Laurencin C.T., Allcock H.R. (2010). Hydrolysable polylactide-polyphosphazene block copolymers for biomedical applications: Synthesis, characterization, and composites with poly(lactic-co-glycolic acid). Polym. Chem..

[206] Deng M., Nair L.S., Nukavarapu S.P., Jiang T., Kanner W.A., Li X., Kumbar S.G., Weikel A.L., Krogman N.R., Allcock H.R. (2010). Dipeptide-based polyphosphazene and polyester blends for bone tissue engineering. Biomaterials.

[207] Veronese F.M., Marsilio F., Lora S., Caliceti P., Passi P., Orsolini P. (1999). Polyphosphazene membranes and microspheres in periodontal diseases and implant surgery. Biomaterials.

[208] Rothemund S., Aigner T.B., Iturmendi A., Rigau M., Husár B., Hildner F., Oberbauer E., Prambauer M., Olawale G., Forstner R. (2015). Degradable Glycine-Based Photo-Polymerizable Polyphosphazenes for Use as Scaffolds for Tissue Regeneration. Macromol. Biosci..

[209] Carampin P., Conconi M.T., Lora S., Menti A.M., Baiguera S., Bellini S., Grandi C., Parnigotto P.P. (2007). Electrospun polyphosphazene nanofibers for in vitro rat endothelial cells proliferation. J. Biomed. Mater. Res. Part A.

[210] Deng M., Kumbar S.G., Nair L.S., Weikel A.L., Allcock H.R., Laurencin C.T. (2011). Biomimetic Structures: Biological Implications of Dipeptide-Substituted Polyphosphazene–Polyester Blend Nanofiber Matrices for Load-Bearing Bone Regeneration. Adv. Funct. Mater..

[211] Peach M.S., Ramos D.M., James R., Morozowich N.L., Mazzocca A.D., Doty S.B., Allcock H.R., Kumbar S.G., Laurencin C.T. (2017). Engineered stem cell niche matrices for rotator cuff tendon regenerative engineering. PLoS ONE.

[212] Narayanan G., Nair L.S., Laurencin C.T. (2018). Regenerative Engineering of the Rotator Cuff of the Shoulder. ACS Biomater. Sci. Eng..

[213] Nukavarapu S.P., Kumbar S.G., Brown J.L., Krogman N.R., Weikel A.L., Hindenlang M.D., Nair L.S., Allcock H.R., Laurencin C.T. (2008). Polyphosphazene/Nano-Hydroxyapatite Composite Microsphere Scaffolds for Bone Tissue Engineering. Biomacromolecules.

[214] Wang S., Wan A.C.A., Xu X., Gao S., Mao H.-Q., Leong K.W., Yu H. (2001). A new nerve guide conduit material composed of a biodegradable poly(phosphoester). Biomaterials.

[215] Xu X., Yee W.-C., Hwang P.Y.K., Yu H., Wan A.C.A., Gao S., Boon K.-L., Mao H.-Q., Leong K.W., Wang S. (2003). Peripheral nerve regeneration with sustained release of poly(phosphoester) microencapsulated nerve growth factor within nerve guide conduits. Biomaterials.

[216] Riva R., Shah U., Thomassin J.-M., Yilmaz Z., Lecat A., Colige A., Jérôme C. (2020). Design of Degradable Polyphosphoester Networks with Tailor-Made Stiffness and Hydrophilicity as Scaffolds for Tissue Engineering. Biomacromolecules.

[217] Müller W.E.G., Tolba E., Schröder H.C., Wang X. (2015). Polyphosphate: A Morphogenetically Active Implant Material Serving as Metabolic Fuel for Bone Regeneration. Macromol. Biosci..

[218] Morozowich N.L., Nichol J.L., Allcock H.R. (2012). Investigation of Apatite Mineralization on Antioxidant Polyphosphazenes for Bone Tissue Engineering. Chem. Mater..

[219] Yang X.-Z., Sun T.-M., Dou S., Wu J., Wang Y.-C., Wang J. (2009). Block Copolymer of Polyphosphoester and Poly(l-Lactic Acid) Modified Surface for Enhancing Osteoblast Adhesion, Proliferation, and Function. Biomacromolecules.

[220] Iwasaki Y. (2020). Bone Mineral Affinity of Polyphosphodiesters. Molecules.

[221] Iwasaki Y., Katayama K., Yoshida M., Yamamoto M., Tabata Y. (2013). Comparative physicochemical properties and cytotoxicity of polyphosphoester ionomers with bisphosphonates. J. Biomater. Sci. Polym. Ed..

[222] Kunomura S., Iwasaki Y. (2019). Immobilization of polyphosphoesters on poly(ether ether ketone) (PEEK) for facilitating mineral coating. J. Biomater. Sci. Polym. Ed..

